# Technological Advances in Anti-hair Loss and Hair Regrowth Cosmeceuticals: Mechanistic Breakthroughs and Industrial Prospects Driven by Multidisciplinary Collaborative Innovation

**DOI:** 10.1007/s00266-025-05077-3

**Published:** 2025-08-11

**Authors:** Xuexue Pan, Rongfei Yu, Jingyi Wu, Wenkai Li, Rongyue Huang, Weiyuan Huang, Yawei Huang, Yingrong Wang, Hualiang Zuo

**Affiliations:** 1 Zhongshan Advanced New Functional Materials Engineering Technology Research Center, Guangdong Engineering Research Center of New Energy Materials and Devices, Zhongshan Polytechnic, Zhongshan, 528400 China; 2 Faculty of Chemical Technology, Poznan University of Technology, Berdychowo 4, 60965 Poznan, Poland; 3 Faculty of Chemistry and Chemical Technology, Al-Farabi Kazakh National University, 71 al-Farabi Ave., 050040 Almaty, Kazakhstan; 4 Institute of Chemical Technology, Faculty of Chemistry, University of Stuttgart, Pfaffenwaldring 55, 70569 Stuttgart, Germany

**Keywords:** Mechanism of hair loss, Anti-hair loss products, Hair growth ingredients, Technological innovation, Market trends

## Abstract

In light of the escalating global prevalence of hair loss, there is an imperative to explore strategies for the prevention and promotion of hair growth. This article reviews the current situation, challenges, innovations, and prospects of cosmetics that promote anti-hair loss and hair growth. Firstly, the physiological and pathological mechanisms of hair loss, including androgenetic alopecia, telogen effluvium, and alopecia areata, as well as the influence of genetic, environmental, and lifestyle factors, are explored. Subsequently, a comprehensive analysis of the predominant product categories and ingredients currently available on the market was conducted, encompassing minoxidil, finasteride, plant extracts, growth factors, and peptides. Building on this, this article further explores the challenges of anti-hair loss and hair growth promotion cosmetics, including effectiveness and safety, consumer acceptance, and the complexity of regulations and standards. This was followed by an introduction to innovations in the field, such as gene therapy, stem cell technology, and microneedling, as well as advanced delivery systems and personalized care options. Finally, this paper looks forward to future technologies’ development trends and market prospects. It emphasizes the importance of multidisciplinary cooperation, including the combination of medicine and cosmetology and the integration of biotechnology and materials science. By synthesizing extant research and delineating prospective research directions, this paper establishes an indispensable reference point for the research and development of cosmetics designed to promote hair growth and prevent hair loss.

*Level of Evidence V* This journal requires that authors assign a level of evidence to each article. For a full description of these Evidence-Based Medicine ratings, please refer to the Table of Contents or the online Instructions to Authors  www.springer.com/00266.

## Introduction

In the contemporary global context, characterized by an aging population, escalating environmental degradation, and mounting life pressures, the prevalence of hair loss has been on the rise on a global scale [1]. The impact of hair loss is twofold, affecting both an individual’s physical appearance and their mental well-being. Indeed, research has demonstrated that hair loss can lead to a decline in self-confidence and social anxiety [2]. Consequently, the demand for hair loss prevention and promotion of new hair growth has increased significantly. There is an increasing demand for practical solutions to prevent hair loss and stimulate new hair growth.

**Fig. 1:**
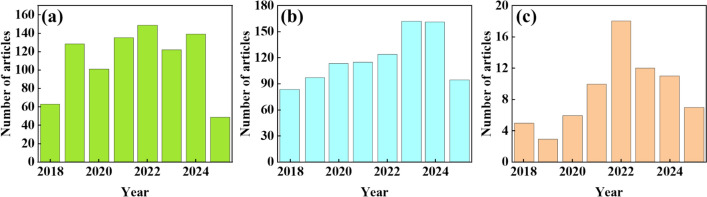
The number of papers published on **a** Cosmeceuticals, **b** Hair regrowth, and **c** Anti-hair loss in Web of Science from 2018 to 2025

According to the analysis of the publication trend of papers based on the Web of Science database from 2018 to 2025, three related research fields show significant differences in development trends. Cosmeceuticals research shows a fluctuating trend of first increasing and then decreasing in Fig. 1a, starting from about 25 articles in 2018, briefly declining to 20 articles in 2020, reaching a peak of about 40 articles in 2021, and then declining year by year. It is expected to drop to around 30 articles by 2025. In contrast, Hair Regrowth has shown sustained strong growth momentum in Fig. 1b, steadily increasing from about 10 articles in 2018, with a significant acceleration in growth rate after 2020, surpassing 40 articles in 2023, and expected to jump to nearly 70 articles by 2025, becoming the fastest growing and highest total research direction among the three fields. Anti Hair Loss research shows the most dramatic fluctuation characteristics, rapidly increasing to about 35 articles from 2018 to 2020 (close to the peak of pharmaceutical cosmetics during the same period) in Fig. 1c, but experiencing a cliff-like decline to a minimum of about 5 articles from 2021 to 2024, and is expected to rebound to around 25 articles by 2025, but still below the level of 2018–2020. Overall, hair growth research is the only continuously growing field, with a significantly higher number of papers than the other two fields since 2024. It is expected to dominate with a scale of about 70 papers by 2025, while pharmaceuticals (about 30 papers) and anti-slip research (about 25 papers) have similar scales but are far lower than the hair growth field. This trend indicates that the study of hair growth mechanisms is rapidly becoming the core direction of academic attention, while the popularity of research on pharmaceuticals and anti-shedding products is showing periodic fluctuations or stage-wise slowing down. This has important guiding significance for grasping future research and development priorities and resource investment.

A wide range of products is available in the market for hair loss prevention and hair growth promotion, spanning from pharmaceutical treatments to cosmetic hair care products [3]. The main product categories include medications such as minoxidil and finasteride and cosmetics containing ingredients like plant extracts, growth factors, and peptides [4]. Although these products show some effectiveness, their efficacy and safety remain controversial [5]. Moreover, consumer acceptance and the complexity of regulations and standards for these products also challenge market development.

This article reviews the current status, challenges, innovations, and prospects of hair loss prevention and hair growth promotion cosmetics. First, the physiological and pathological mechanisms of hair loss will be discussed, including androgenetic alopecia, telogen effluvium, and alopecia areata, along with an analysis of the impact of genetic, environmental, and lifestyle factors [6]. Next, the main product categories and ingredients in the current market will be analyzed, evaluating their effectiveness and safety. The article will then explore the key challenges hair loss prevention and hair growth cosmetics face, including consumer acceptance and the complexity of regulatory standards. Future innovations in the field will be introduced, such as gene therapy, stem cell technology, microneedling, advanced delivery systems, and personalized care solutions. Finally, the article will forecast the future development trends of technologies and market prospects, emphasizing the importance of multidisciplinary collaboration to provide valuable references for researching and developing hair loss prevention and growth promotion cosmetics.

## The Mechanisms and Influencing Factors of Hair Loss

### Physiological and Pathological Mechanisms of Hair Loss

**Fig. 2:**
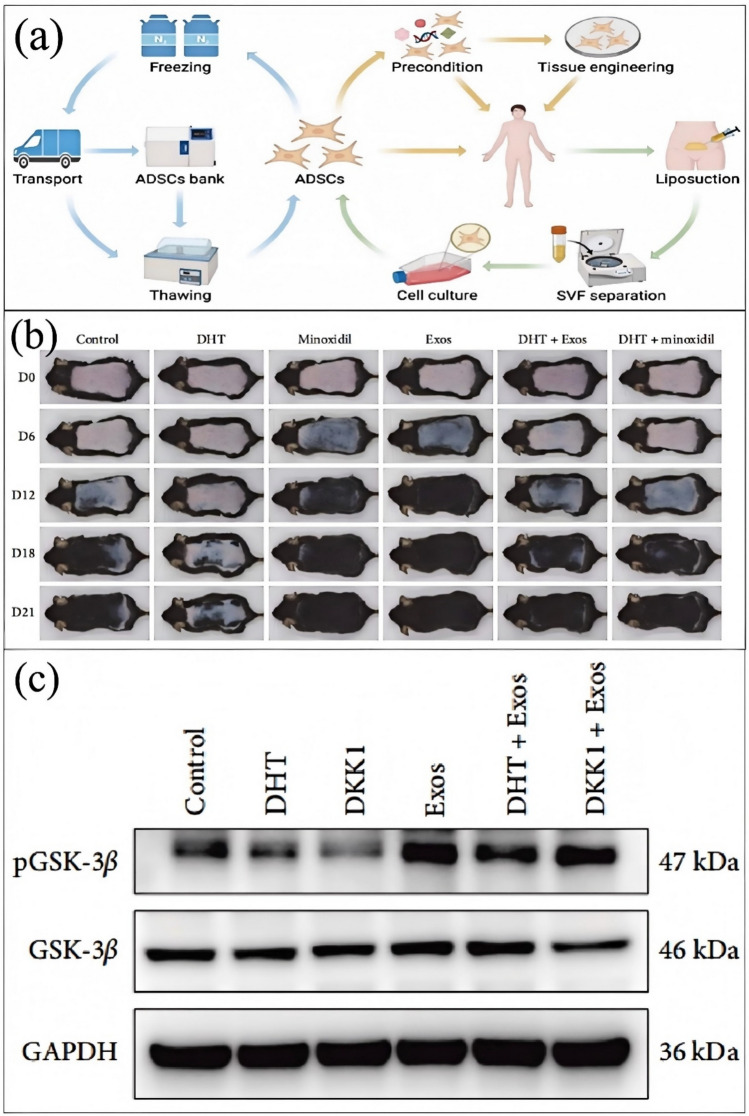
**a** Procedures for applying adipose-derived stem cells (ADSCs). Green arrows indicate the isolation of ADSCs [7]. **b** ADSC-Exos increased skin thickness and the number of hair follicles and accelerated telogen-to-anagen transition. **c** ADSC-Exos antagonizes the inhibitory effect of DHT by activating the Wnt/β-catenin pathway. ADSC-Exos partially reverses the inhibitory effect of DHT on pGSK-3β [9]

Hair loss is a complex physiological and pathological phenomenon, with mechanisms involving the interplay of various internal and external factors. The causes and mechanisms of hair loss are multifaceted, including genetics, hormone levels, immune regulation, cytokines, growth factors, stress, and other factors. The main types of hair loss include androgenetic alopecia, alopecia areata, and chemotherapy-induced hair loss. Treatments for hair loss are also continuously advancing, including pharmaceutical treatments, adipose-derived stem cells (Fig. 2a) and their derivatives, and the application of traditional Chinese medicine and its active ingredients [7].

Androgenetic Alopecia (AGA) is the most common type of hair loss, and its pathogenesis may be influenced by the interaction of multiple genetic and environmental factors [8]. Studies have shown that adipose-derived stem cells and their derivatives, due to their ability to regulate the hair follicle cycle (Fig. 2b), improve the scalp microenvironment, and antagonize androgens (Fig. 2c), have significant research value [9]. Additionally, research on the use of the herbal treatment Wubao Hair Growth Tincture, based on the HGF/c-Met signaling pathway for treating androgenetic alopecia, has shown that this method can balance hormones by activating the HGF/c-Met signaling pathway, thus treating androgenetic alopecia.

Traditional Chinese medicine (TCM) and its active ingredients also show potential for hair loss prevention and growth promotion [10]. For example, research on Wubao Hair Growth Tincture has demonstrated that using fresh cedar leaves as raw materials is more effective than dry leaves. Furthermore, polyunsaturated fatty acids have been proven to promote or inhibit the release of signaling molecules from hair follicle cells, thereby promoting hair follicle growth, improving the hair follicle cycle, and aiding in follicle regeneration [11].

**Fig. 3:**
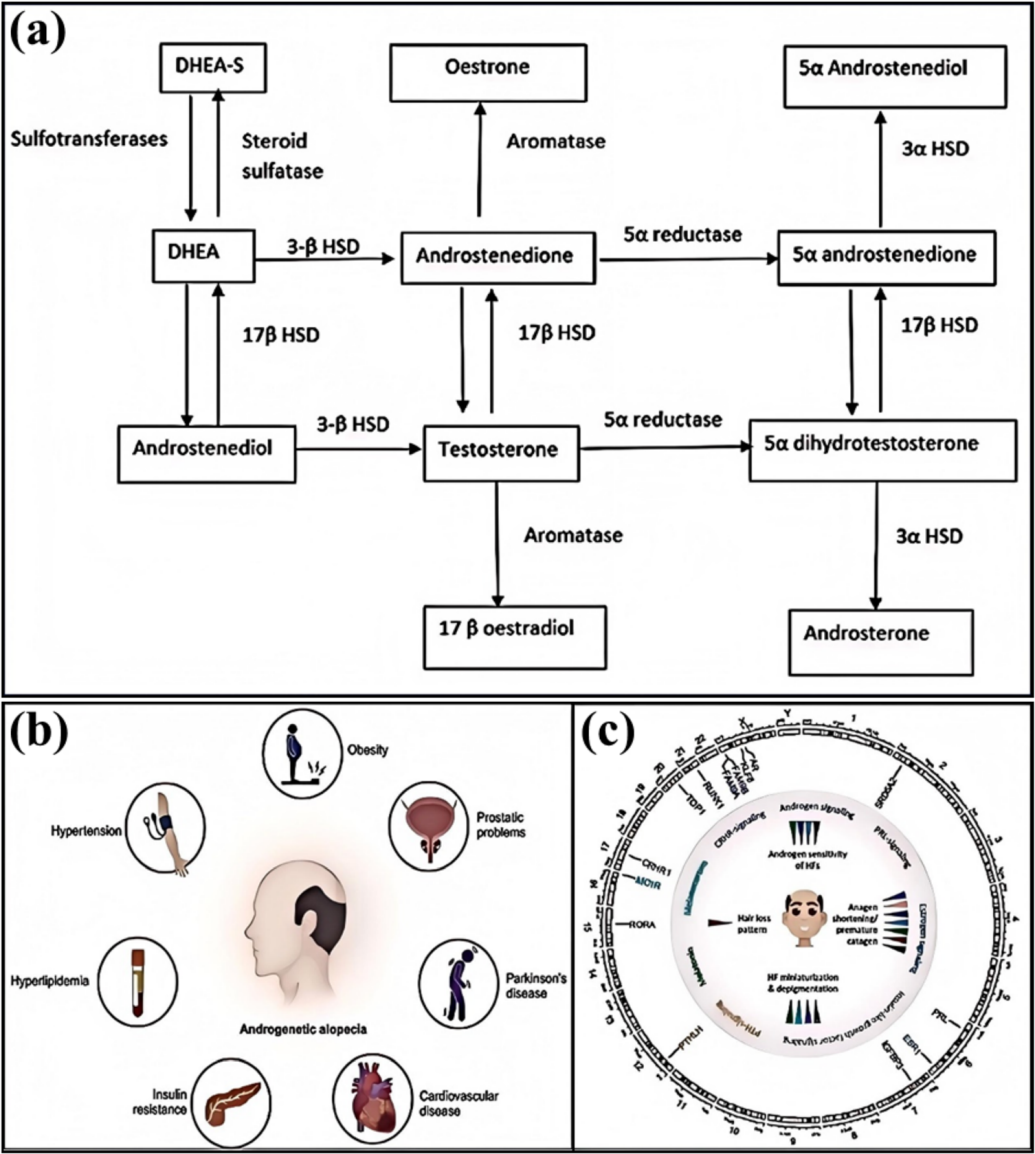
**a** Androgen metabolism in the skin [12]. **b** Potential conditions associated with early-onset AGA [15]. **c** Genetic pointers to the role of (sex) hormones in the development of AGA [13]

Hair loss treatment must be customized according to its specific causes and types. For example, androgenetic alopecia can be treated with topical minoxidil and oral finasteride. At the same time, studies on traditional Chinese medicine hair growth solutions in mice suggest that TCM hair growth solutions have efficacy in promoting hair growth. Additionally, for non-physiological hair loss, such as immune-deficient alopecia (IDA) and traumatic alopecia (SDA), their pathological processes are more frequently reported. AGA results from hair follicle sensitivity to androgens, disrupting the growth cycle (Fig. 3a) [12]. At the same time, IDA involves the collapse of the hair follicle immune tolerance mechanism, where immune cells attack the hair follicles.

Therefore, treating hair loss requires a comprehensive consideration of its complex etiology and mechanisms, utilizing multidimensional and multi-angle therapeutic approaches. Future research should continue to explore more effective treatment methods to meet the needs of patients.

#### Androgenetic Alopecia

Androgenetic Alopecia (AGA) is a common type of hair loss, and its pathogenesis involves both genetic factors and the action of androgens. The development of AGA is closely associated with the role of androgens (Fig. 3c), particularly dihydrotestosterone (DHT) [13]. DHT binds to androgen receptors in the hair follicles, causing them to shrink gradually, shortening the anagen phase, and ultimately leading to hair thinning and shedding [14]. Furthermore, the onset of AGA is also influenced by genetic factors, with studies indicating that genetic variations in the androgen receptor gene (AR) in humans are the primary determinants for the development of early-onset AGA (Fig. 3b) [15].

AGA manifests differently in males and females [16]. In males, AGA typically presents as hair loss at the forehead and crown areas, while in females, it appears as diffuse thinning on the top of the scalp. This gender difference may be related to the expression levels of androgen receptors and associated enzymes in the hair follicles [17]. Studies have shown that in females, the levels of androgen receptors and 5α-reductase I and II in the frontal hair follicles are lower than in males, while the level of aromatase is higher in females [18]. These differences may be one of the reasons for the distinct clinical presentations of AGA in males and females.

Genetic studies have further revealed the complexity of AGA. Although several gene loci associated with AGA have been identified, most genetic risk factors remain unknown [19]. Additionally, the pathogenesis of AGA involves not only the androgen receptor signaling pathway but may also involve other hormonal and molecular signaling pathways [20]. This suggests that the pathogenesis of AGA may be more complex than currently understood.

AGA treatment options include pharmaceutical, surgical, and non-surgical treatments Currently [21], only two medications—topical minoxidil and oral finasteride—are approved by the U.S. Food and Drug Administration (FDA) and the European Medicines Agency (EMA) for the treatment of AGA [22]. However, due to AGA’s polygenic nature and complex pathogenesis, finding new treatment methods remains challenging.

Therefore, AGA is a complex hair loss disorder, with its pathogenesis involving both genetic factors and the action of androgens. While some progress has been made, a comprehensive understanding of AGA remains limited, and further research is needed to uncover its complex genetic background and pathogenesis.

#### Telogen Effluvium

Telogen Effluvium (TE) is a common type of hair loss characterized by the sudden shedding of a large number of hairs that are typically in the telogen (resting) phase [23]. This type of hair loss can be acute or chronic and is typically associated with external factors such as physiological or psychological stress, malnutrition, illness, medication side effects, and hormonal changes [24]. The diagnosis of TE primarily relies on patient history and clinical presentation, and sometimes, a scalp biopsy is necessary to rule out other types of hair loss [25].

Acute Telogen Effluvium (TE) typically occurs within 3–4 months after a triggering event, presenting as diffuse hair loss, and the hair can fully regrow within several months after the removal of the trigger [26]. Chronic TE, on the other hand, manifests as persistent, unexplained hair loss, and recovery may take longer. The key to treating TE is identifying and removing the trigger; medications such as corticosteroids and minoxidil can also be used for treatment [27].

TE differs from other types of hair loss, such as Androgenetic Alopecia (AGA) and Alopecia Areata. AGA usually presents with a specific pattern of hair loss, while Alopecia Areata results in patchy hair loss. TE typically causes diffuse shedding; on dermoscopy, an increase in vellus hairs and a reduction in pigmentation can be observed [28].

In terms of treatment, non-pharmacological methods such as psychological and nutritional support can be employed in addition to pharmacological approaches. Furthermore, some studies have explored the potential effectiveness of supplements like lactoferrin in treating chronic TE.

Therefore, TE is a type of hair loss associated with multiple factors, and its treatment requires a comprehensive approach that considers the patient’s specific situation, including identifying and removing triggers and appropriate pharmacological and non-pharmacological interventions.

#### Alopecia Areata

Alopecia Areata (AA) is an autoimmune disorder characterized by the appearance of round or oval-shaped hair loss patches on the scalp or other areas of the body [29]. The exact pathogenesis of this disease is not yet fully understood, but it is closely related to genetic and environmental factors. The pathogenesis, clinical presentation, diagnostic methods, and treatment progress of Alopecia Areata can be explored from several perspectives.

##### Pathogenesis

The pathogenesis of Alopecia Areata involves multiple factors, including genetics, immune system abnormalities, and environmental factors. Genetic factors play an essential role in the development of AA. Studies show that some patients with Alopecia Areata have a family history, indicating that genetics contribute significantly to its occurrence. Additionally, abnormal immune responses are a key mechanism in the development of AA. Alopecia Areata is considered an autoimmune disease, where the immune system mistakenly attacks hair follicles, causing them to enter the telogen phase and resulting in hair loss [30]. In particular, CD8+ NKG2D+ T cells dominate follicular pathology, although the exact mechanism remains unclear [31].

##### Clinical Presentation and Diagnosis

The clinical presentation of Alopecia Areata varies, ranging from a single hair loss patch to total hair loss on the body [32]. Most patients develop symptoms before the age of 30, and the clinical types of AA include patchy, reticulate, and diffuse forms. The diagnosis of Alopecia Areata is typically based on clinical signs, such as non-scarring, round hair loss patches, and the early-stage “exclamation mark” hairs [33]. In more complex or unusual cases, biopsy and histological examination may be used to assist in the diagnosis.

##### Treatment Progress

Although Alopecia Areata has no cure, treatment methods have progressed [34]. Traditional treatments include topical or systemic corticosteroids, local immunotherapy, and topical hair growth stimulants [35]. In recent years, new immunotherapy drugs, such as small molecule drugs and biologics, have also shown promise [36]. However, the effectiveness of these treatments is inconsistent, and treatment interruptions often lead to relapse of Alopecia Areata.

Alopecia Areata is a complex autoimmune disease with pathogenesis involving genetics, immune system abnormalities, and environmental factors. Despite some progress in current treatments, many challenges remain, such as the inconsistency in treatment outcomes and high relapse rates. Future research needs to clarify the specific mechanisms of Alopecia Areata further to develop more effective and safer treatment options.

#### Unresolved Controversies and Debates in Hair Loss Pathophysiology

Multiple pieces of evidence indicate the presence of “microinflammation” around hair follicles in AGA patients, manifested as infiltration of lymphocytes and mast cells, and elevation of pro-inflammatory cytokines (IL-6, TNF-α, TGF-β1) [37, 38]. This inflammation may be triggered by metabolic products of scalp microbiota (such as *Propionibacterium acnes*) or oxidative stress, leading to follicular fibrosis and thickening of the collagen sheath, which accelerates follicular miniaturization [37, 39]. Additionally, androgens may directly induce inflammation by activating innate immune responses (such as the TLR pathway) [38]. Some studies suggest that inflammation is a consequence rather than a cause of follicular degeneration. For example, R. English et al. point out that the heterogeneity of biopsy sites and hair follicle miniaturization stages (HFM) may lead to biases in inflammation detection results and the degree of inflammation may not be directly correlated with hair loss progression [40]. Furthermore, although finasteride inhibits DHT production, it does not significantly improve inflammatory markers, suggesting that inflammation may be a secondary event [41, 42]. Longitudinal studies are needed to clarify the temporal relationship between inflammation and AGA, and single-cell transcriptomics should be used to analyze the subtypes of inflammatory cells and signaling pathways [37, 40].

Dysregulation of the Wnt/β-catenin Pathway is crucial for hair follicle cycle regulation, and DHT may cause follicular miniaturization by inhibiting Wnt signaling [43]. However, Wnt activators (such as valproate) have shown limited efficacy in clinical trials, suggesting their effects may be interfered with by other factors [44]. Scalp follicles in AGA patients exhibit mitochondrial dysfunction and abnormal glucose metabolism (such as reduced HIF-1α and insufficient glycogen storage), leading to inadequate energy supply [39, 43, 45]. Oxidative stress can also activate TGF-β1 and promote fibrosis [42]. The imbalance between PGD2 (inhibiting hair growth) and PGE2 (promoting growth) may affect hair follicles independently of DHT [44], but clinical validation remains insufficient. Although 30–40% of patients show poor response to finasteride, whether DHT-independent mechanisms have independent pathogenicity remains unclear [46]. For example, Cuevas-Diaz Duran et al. show that DHT levels are not directly correlated with the severity of hair loss, suggesting that follicular sensitivity to androgens (such as AR gene polymorphisms) may be more critical [43]. Genetic/epigenetic analyses should be integrated to identify subgroups that benefit from DHT-independent therapies [39, 43].

Topical melatonin and polyphenolic compounds can improve follicular function by reducing oxidative stress (such as inhibiting ROS) [47, 48]. JAK inhibitors (such as tofacitinib) are effective in alopecia areata but show inconsistent effects in AGA, possibly related to differences in inflammation types [38, 47]. Small-scale trials have publication bias, and the placebo effect is significant. For example, Natarelli et al. indicate that the reliability of meta-analysis results on the efficacy of antioxidants is insufficient [47]. Additionally, inflammation in AGA may exhibit “dynamic stage dependence,” such as Th1 cell dominance in the early stage and fibrosis as a characteristic in the late stage, requiring targeted interventions [40]. Dose-response trials should be conducted, using standardized endpoints (such as the vellus/terminal hair ratio) to evaluate efficacy, and non-invasive inflammatory markers (such as scalp cytokine detection) should be developed [40].

### The Impact of Genetic, Environmental, and Lifestyle Factors

**Fig. 4:**
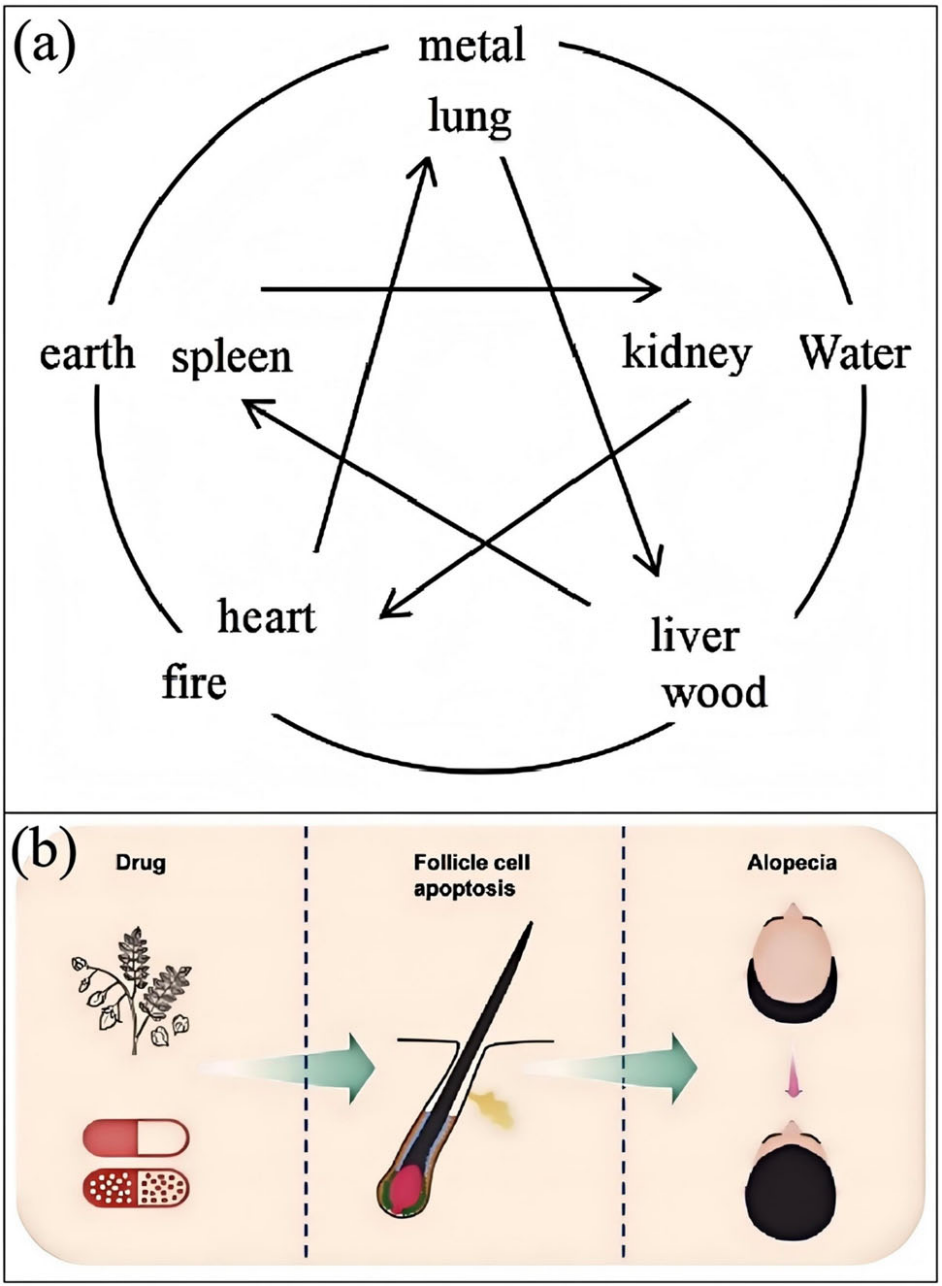
**a** The diagram of five elements generating and suppressing each other [50]. **b** Relationship between ingredients, apoptosis, and alopecia. This article introduces the process of cell apoptosis and the cycle of hair follicles, focusing on elucidating the apoptosis that occurs in the hair follicle [55]

Hair loss is a complex medical issue, with causes involving multiple factors, including genetics, endocrine factors, the immune system, lifestyle, and nutrition [49]. From a Traditional Chinese Medicine (TCM) perspective, hair loss is closely related to the kidneys, liver, and spleen, with complex etiological and pathological mechanisms (Fig. 4a) [50]. Western medicine, conversely, considers hair loss to be the result of the combined effects of multiple factors, including infections, hormone levels, immune regulation, cytokines, growth factors, stress, and genetics [51]. These viewpoints suggest that a single factor does not cause hair loss but results from multiple factors.

Specifically, studies on Androgenetic Alopecia (FAGA) show that family history, frequent late nights, sleep disorders, a preference for high-sugar foods, a high-fat diet, significant mental stress, dieting, and the presence of other diseases are independent risk factors for FAGA in young individuals. This confirms the relationship between hair loss, lifestyle, and dietary habits [52]. In addition, the causes of hair loss include immune dysfunction, skin diseases, environmental factors, nutrition-related hair loss, and medication-induced hair loss [53].

Research on Alopecia Areata also indicates that genetics, immunity, mental and psychological factors, vitamins and trace elements, metabolism, and microorganisms can all contribute to the condition’s onset [54]. The combined effects of these factors lead to the occurrence of Alopecia Areata, highlighting the multifactorial nature of hair loss.

Therefore, hair loss is a complex phenomenon caused by the interaction of various factors (Fig. 4b) [55]. These factors include, but are not limited to, genetics, endocrine imbalances, immune system abnormalities, lifestyle and dietary habits, and nutritional status. Therefore, treating hair loss requires a comprehensive approach that considers these factors, employing multiple therapeutic measures.

#### Genetic Factors

**Fig. 5:**
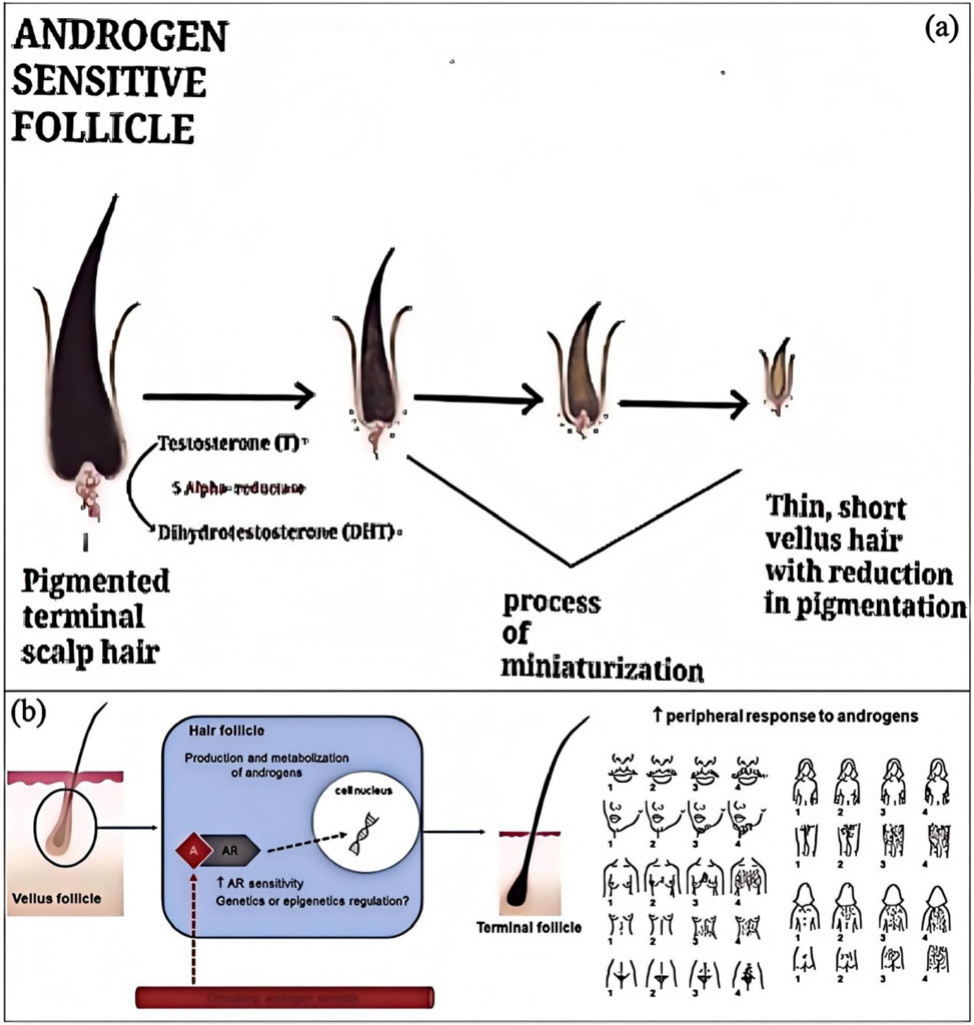
**a** Pathogenesis of AGA [56]. **b** Role of androgens in the hair follicle and its peripheral response [57]

Genetic factors play a crucial role in Androgenetic Alopecia (AGA) (Fig. 5a) [56]. AGA, also known as male pattern baldness, is the most common form of hair loss in humans, and its pathogenesis is closely related to genetic predisposition and androgen levels. Studies show that the occurrence of AGA is associated with variations in multiple genes, with polymorphisms in the Androgen Receptor (AR) gene being considered one of the main determinants of AGA. Additionally, family history is a significant risk factor for AGA, as individuals with a family history of hair loss are more likely to experience it themselves.

Polymorphisms in the Androgen Receptor (AR) gene significantly influence an individual’s sensitivity to androgens, thus affecting the onset and progression of hair loss (Fig. 5b) [57]. For example, variations in the length of the GGN repeat sequence in the AR gene are associated with the risk of AGA, with individuals having ≤ 23 repeats exhibiting a significantly increased likelihood of hair loss. This suggests that polymorphisms in the AR gene may contribute to the pathogenesis of AGA by influencing an individual’s sensitivity to androgens.

In addition to the AR gene, other genes also play a role in the pathogenesis of AGA. For instance, the discovery of new susceptibility gene loci on chromosomes 3q26 and 20p11 indicates that non-androgen-dependent pathways are also involved in the development of AGA. Furthermore, several gene loci associated with AGA have been identified, including those encoding HDAC4, HDAC9, and the WNT molecule WNT10A. These findings provide new insights into the genetic foundation of AGA.

The role of family history in the onset of AGA has also been extensively studied [58]. Research shows that males with a family history of hair loss are more likely to experience hair loss compared to those without such a family history. This highlights the critical role of genetic factors in the development of AGA, with paternal inheritance being more likely to lead to hair loss than maternal inheritance. Additionally, family history is associated with the type, onset age, and severity of hair loss [59].

Genetic factors play a crucial role in Androgenetic Alopecia (AGA). Polymorphisms in the Androgen Receptor (AR) gene, family history, and variations in multiple gene loci are all closely associated with the onset and progression of AGA. These findings provide important insights into the genetic mechanisms of AGA and offer potential directions for developing new treatment strategies.

#### Environmental Factors

Environmental factors have a multifaceted impact on the scalp and hair follicles, including air pollution, ultraviolet (UV) radiation, and chemical exposure. These factors influence hair health and growth through various mechanisms [60].

**Fig. 6:**
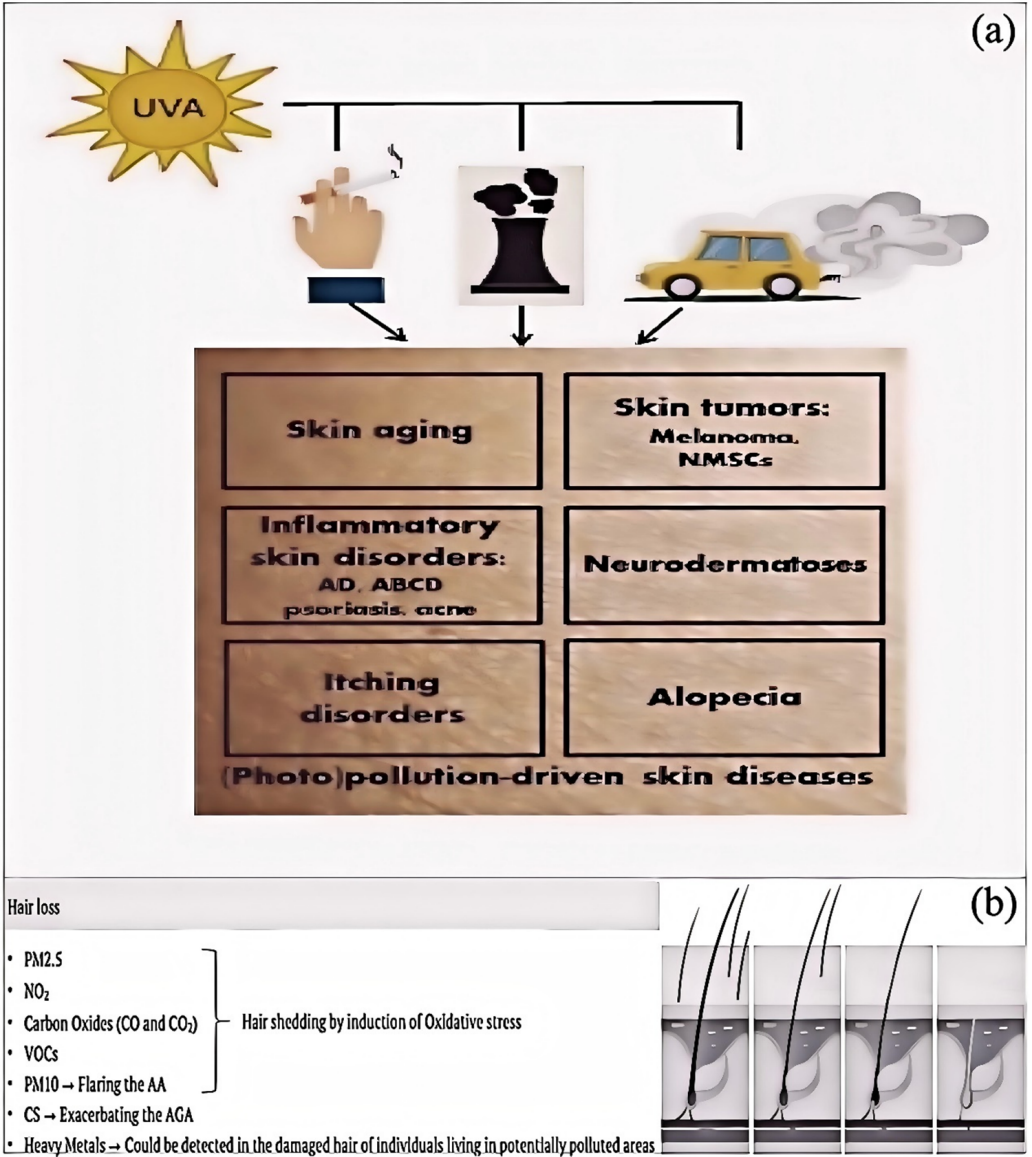
**a** The deleterious synergic effect of UVA and air pollution on the skin [67]. **b** Mechanisms of air pollution and hair damage [61]

Air pollution, especially particulate matter (such as PM10), has been shown to reduce the levels of proteins that promote hair growth on the scalp, thus promoting hair loss (Fig. 6b) [61]. In addition, airborne pollutants such as tobacco smoke and phthalates, which are endocrine disruptors, may also indirectly affect the function of hair follicles [62].

UV radiation is another significant environmental factor that affects hair follicles’ physical structure and physiological functions and induces oxidative stress and changes in the micro-ecology, leading to inflammation around the hair follicles. UV exposure can also cause hair follicle stem cells and dermal papilla cells to age, further promoting hair loss [63].

Exposure to chemicals, such as ingredients in hair dyes and styling products, is another important factor leading to hair damage [64]. These chemicals can damage the structure and function of hair, making it brittle, prone to breakage, or causing hair loss [65]. For instance, the chemicals in hair dyes can cause melanin deposition in the cuticle and cortex, which provides some protection against UV light but may negatively affect hair health with long-term use [66].

Environmental factors affect the health of the scalp and hair follicles through various mechanisms (Fig. 6a, b), including direct physical and chemical damage and indirect endocrine and oxidative stress effects [67]. Therefore, to maintain healthy hair, it is recommended to take appropriate protective measures, such as reducing exposure to polluted air and UV radiation and using hair care products containing harmful chemicals with caution [68].

#### Lifestyle Factors

Unhealthy lifestyles, such as poor eating habits, lack of exercise, smoking, and excessive alcohol consumption, can indeed harm hair health. Nutritional deficiencies, particularly the lack of essential nutrients like iron, zinc, vitamin D, and protein, can lead to fragile hair and hair loss [69]. Stress and insufficient sleep can also trigger or exacerbate hair loss issues [70].

Multiple studies have confirmed that nutritional factors significantly impact hair health. For example, iron deficiency is associated with hair loss in women, with serum ferritin concentration a good indicator of an individual’s iron status [71]. Additionally, essential amino acids like L-lysine have been important in preventing hair loss [72]. However, it is crucial to note that excessive intake of nutritional supplements may lead to hair loss rather than improvement [73].

Lifestyle changes are equally important for preventing hair loss [74]. For example, adolescent hair loss is often linked to unhealthy habits, such as poor diet, staying up late, emotional fluctuations, lack of exercise, and frequent use of hair dyes and heat treatments [75]. Therefore, it is recommended that adolescents adopt healthier habits to reduce the occurrence of hair loss.

Furthermore, oxidative stress is a key factor contributing to hair loss, and nutrients can combat oxidative stress, repair cellular damage, support cell function, and promote hair regrowth [76]. This suggests an appropriate nutritional environment can neutralize free radicals and maintain an active hair growth cycle.

Therefore, unhealthy lifestyles and poor nutrition are significant factors leading to fragile hair and hair loss [77]. Improving dietary habits, increasing physical activity, reducing stress, and ensuring adequate sleep can effectively prevent and alleviate hair loss issues. At the same time, proper nutritional supplementation is necessary, but care should be taken to avoid excessive intake of nutrients that may cause adverse effects [78]. Therefore, hair loss results from complex physiological and pathological mechanisms influenced by various genetic, environmental, and lifestyle factors. Understanding these factors will help develop more effective solutions for preventing and promoting hair regrowth.

### Demographic Variations in Hair Loss Patterns and Pathophysiology

#### Ethnic Differences in Androgen Receptor Expression and Hair Loss

The influence of ethnic differences in androgen receptor (AR) gene expression on the incidence of androgenetic alopecia (AGA) primarily manifests in genetic polymorphisms, enzymatic activity, and gene-environment interactions, as analyzed below:

1. Caucasian Populations (European Descent)
  **Shorter CAG Repeat Sequences**: The number of CAG repeats in the AR gene among Caucasians is typically fewer than 22. Shorter CAG repeats are associated with enhanced AR transcriptional activity, leading to increased hair follicle sensitivity to dihydrotestosterone (DHT). This genetic feature significantly elevates the risk of AGA, with an incidence rate as high as 30–50%. **Haplotype and Positive Selection**: The Eur-H1 haplotype, strongly associated with AGA, is present in European populations. This haplotype covers the AR and *EDA2R* gene regions and exhibits characteristics of linkage disequilibrium and selective sweep [79]. For example, the R57K mutation (rs1385699) in the *EDA2R* gene is highly prevalent in European and East Asian populations, potentially influencing AGA risk by enhancing signaling pathways related to hair follicle development. **Environmental Factors**: A Western high-fat diet may exacerbate follicular atrophy by upregulating lipocalin-type prostaglandin D2 synthase (L-PGDS), further increasing the incidence rate.
2. East Asian Populations (e.g., China, Korea, Japan)
  **Longer CAG Repeat Sequences**: The average number of CAG repeats in East Asian populations is higher than that in Europeans. Longer repeats may reduce AR activity, partially explaining the lower AGA incidence (15–20%) in this group. **Role of Specific SNPs**: Despite longer CAG repeats, a Korean study showed significant enrichment of the rs6152 SNP (G allele) in the AR gene among AGA patients, suggesting that other genetic variants (such as non-coding region SNPs) may supplement risk regulation. Additionally, although the frequency of the Eur-H1 haplotype is high in East Asians, the AGA incidence remains lower than in Europeans, possibly due to gene-environment interactions or differences in epigenetic regulation[79].
3. African Populations
  **Unique Promoter Haplotypes**: Unique haplotypes (e.g., rs12014709-T) in the AR gene promoter region among Africans may suppress AR expression and reduce hair follicle sensitivity to DHT. Furthermore, lower activity of 5α-reductase type II (SRD5A2) in Africans further decreases DHT production, resulting in an AGA prevalence of only 5–10%. **Genetic Diversity**: Lower levels of linkage disequilibrium in the AR/*EDA2R* region, with more dispersed genetic variations, may weaken the effects of AGA-associated risk alleles [79].

Some studies indicate no independent association between CAG repeat length and AGA, while the number of GGC repeats (e.g., ≥23) may more significantly influence AGA risk and treatment response [8, 80]. For example, individuals with higher GGC repeat counts show poorer responses to finasteride [80]. The AR gene accounts for only ~40% of genetic risk. Racial differences in other genes (e.g., *EDA2R*, *WNT10A*) and epigenetic regulation (e.g., AR promoter methylation) require further investigation [81, 82].

#### Sex-Specific Patterns of Alopecia

The sex-specific patterns of androgenetic alopecia (AGA) are mainly reflected in two aspects: differences in androgen/estrogen balance and differences in the hair follicle stem cell microenvironment, which are analyzed as follows:

1. Sex Differences in Androgen/Estrogen Balance
  Male AGA is primarily driven by type II 5α-reductase (SRD5A2), an enzyme that converts testosterone into the more active dihydrotestosterone (DHT). DHT inhibits the Wnt/β-catenin signaling pathway in dermal papilla cells by binding to androgen receptors (AR), leading to hair follicle miniaturization and shortened anagen phase [83–85]. The expression level of SRD5A2 in the frontal and vertex hair follicles of males is 3-5 times higher than that in the occipital region, which explains the selectivity of alopecia areas [86]. Additionally, DHT further suppresses hair follicle regeneration through the PGD2-CXXC5 axis, forming a vicious cycle [84].
  The activity of SRD5A2 in female hair follicles is only 40% of that in males, and the AR content in frontal hair follicles is 40% lower than in males [86]. Meanwhile, the content of aromatase (which converts testosterone into estrogen) in female hair follicles is six times higher than in males, and estrogen protects hair follicles by prolonging the anagen phase [43, 86]. After menopause, the decline in estrogen levels leads to relative androgen dominance, causing female AGA to typically manifest as diffuse vertex thinning rather than the typical male pattern of frontal hairline recession [43, 87, 88].
2. Differences in Hair Follicle Stem Cell Microenvironment
  The retention rate of hair follicle stem cell markers (such as LGR5 and KRT15) in female AGA patients is significantly higher than in males, which may explain the slower progression of hair loss in females [85, 89, 90]. Animal models have shown that androgens can induce apoptosis of male hair follicle stem cells, while female stem cells exhibit stronger resistance to androgens, possibly related to estrogen-mediated protective mechanisms [43, 85]. Early-stage male AGA is characterized by hair diameter deterioration, followed by a reduction in hair unit numbers; in females, hair unit numbers decrease first, with diameter thinning occurring later [90]. This difference may be associated with sex-specific regulation of stem cell responses to androgens [85, 90].
3. Controversies and Unresolved Questions
  The diagnostic criteria for female AGA remain unstandardized. Some patients have normal androgen levels (“idiopathic AGA”), suggesting that non-androgenic pathways (such as insulin resistance, iron deficiency, and inflammation) may be involved in pathogenesis [43, 88, 91]. Thickened scalp subcutaneous fat in metabolic syndrome patients may activate 5α-reductase through mechanical pressure, exacerbating alopecia [89, 92]. 5α-reductase inhibitors such as finasteride are highly effective for male AGA but have limited efficacy in females, possibly due to the multifactorial pathological mechanisms of female hair loss [43, 88, 91]. Emerging therapies such as PRP injection and microneedling still require more sex-stratified studies for validation [43, 93].

#### Hormonal Factors in Postmenopausal and Postpartum Populations

The Dual Hormonal Mechanism of Postmenopausal Hair Loss Estrogen Withdrawal Effect: Postmenopausal serum estradiol levels significantly decline (from 30–400 pg/mL during the reproductive period to < 20 pg/mL), removing the inhibitory effect on hair follicle androgen receptors (AR) and leading to activation of the dihydrotestosterone (DHT) signaling pathway [94]. Estrogen binds to ER-β receptors in hair follicles and inhibits the conversion of androgens to DHT by regulating aromatase activity; a decrease in estrogen levels directly exacerbates the negative effects of androgens on hair follicles [88, 94].

Androgen Metabolism Imbalance: Although ovarian secretion of androstenedione decreases, the conversion rate of adrenal dehydroepiandrosterone (DHEA) to testosterone increases. Combined with a reduction in sex hormone-binding globulin (SHBG) concentration, this results in a relative increase in free testosterone levels [94, 95]. Elevated postmenopausal luteinizing hormone (LH) levels further stimulate ovarian stromal cells to secrete androgens, creating a local microenvironment of hyperandrogenism [96]. Notably, this estrogen/androgen ratio imbalance not only affects the hair follicle cycle but also forms a vicious cycle with insulin resistance and abdominal fat accumulation [95, 97].

Heterogeneous Recovery of Postpartum Hair Loss (Telogen Effluvium) Hormonal Fluctuation Trigger Mechanism: High estrogen levels during pregnancy inhibit normal hair shedding by prolonging the anagen phase of hair follicles. The abrupt decline in estradiol and progesterone after childbirth triggers synchronous entry of hair follicles into the telogen phase. Approximately 90% of postpartum women experience diffuse hair loss at 3–4 months after delivery, but recovery trajectories exhibit significant variability [94, 98]: (1) **Complete Recovery (approximately 60%)**: Predominantly observed in younger women (< 35 years old) without a genetic predisposition to androgenetic alopecia (AGA), hair density is restored within 6–12 months, possibly associated with low follicular sensitivity to androgens and adequate iron reserves. (2) **Incomplete Recovery (approximately 30%)**: Women with underlying AGA or ferritin < 40 μg/L are prone to developing chronic telogen effluvium, characterized by persistent hair thinning. The mechanism involves an increase in the proportion of miniaturized follicles and growth cycle disorders [94, 98].

The impact of breastfeeding on postpartum hair loss remains controversial: Some studies propose that prolactin prolongs the hair recovery cycle by inhibiting estrogen synthesis via the hypothalamic-pituitary-gonadal axis [98], but a recent meta-analysis found no significant correlation between prolactin levels and Hair loss severity. This discrepancy may arise from differences in research methodologies, such as whether confounding factors like thyroid function and iron metabolism were controlled for. Additionally, individual variations in androgen receptor polymorphisms, local follicular 5α-reductase activity, and other factors have not been fully incorporated into analyses [86, 94], necessitating further multicenter cohort studies to clarify their regulatory networks.

#### Pigment-Dependent Safety of Treatments

Laser treatments (such as low-energy laser therapy) generate thermal effects through the selective absorption of light energy by melanin. However, Fitzpatrick IV-VI skin types exhibit higher epidermal melanin content, leading to significantly enhanced photothermal conversion efficiency, which may trigger epidermal thermal injury complications. These manifest specifically as blister formation and post-inflammatory hyperpigmentation (PIH), with mechanisms involving the activation of melanocytes by inflammatory mediators such as prostaglandins and cytokines, promoting melanin synthesis and transfer to keratinocytes [99–101]. It is noteworthy that although some studies suggest no significant correlation between PIH incidence and skin type [99], most clinical observations indicate a higher risk of PIH in dark-skinned populations, with more stubborn symptoms [100–102]. Prioritize long-wavelength lasers such as 1064 nm Nd:YAG, which have a lower melanin absorption coefficient (approximately 3–10 times lower than the 532 nm wavelength). This allows penetration into the dermis while reducing epidermal energy accumulation, making them suitable for treating IV-VI skin types [103–105]. Extend the pulse duration to the 1–10 ms range to reduce the risk of instantaneous thermal injury by matching the thermal relaxation time (TRT) of melanin granules; combined with dynamic cooling techniques (such as contact cooling or cold spray systems), this can further protect the epidermal barrier [103, 106]. Adopt a “low-fluence, multiple-pass” mode, such as controlling the energy density within the 3.0–3.8 J/cm^2^ range and administering 5–8 treatments, to balance efficacy and safety [105, 107]. Take minoxidil as an example: the incidence of contact dermatitis in African descent populations (12%) is significantly higher than in Caucasian populations (5%). Possible mechanisms include: ① increased permeability of the propylene glycol vehicle due to differences in epidermal barrier function; ② prolonged drug retention time caused by curly hair follicle morphology; ③ enhanced sensitivity of melanocytes to irritating components [102, 103]. Such differences suggest the need to adjust drug carriers (such as switching to propylene glycol-free formulations) and administration frequency according to ethnicity. Most existing laser systems are designed based on the optical properties of Fitzpatrick I–III skin types, lacking customized parameter algorithms for the melanin distribution characteristics of IV–VI skin types, which leads to deviations in efficacy prediction models [104, 108]. Most clinical trials focus on East Asian populations, with a scarcity of high-quality multicenter studies on African or South Asian populations, particularly lacking long-term safety data on new technologies such as picosecond lasers and fractional photothermal therapy in dark skin [104, 108, 109]. Preoperative topical hydroquinone (2–4%), postoperative broad-spectrum sunscreens (containing zinc oxide/titanium dioxide), and anti-inflammatory ingredients (such as dipotassium glycyrrhizinate) can reduce the risk of PIH, but the optimal intervention timing and dosage still require standardization [100, 110].

## The Current Status of Hair Loss Prevention and Hair Growth Cosmetics

### Existing Product Categories

The anti-hair loss and hair regrowth cosmetics market has diversified in recent years, with various products designed to address different causes of hair loss and meet consumer needs. The main types of products can be summarized as follows:

1. Hair Growth Shampoos: These products target mild hair loss issues and aim to improve hair health by adding ingredients that prevent hair loss, strengthen hair, and reduce shedding. For example, shampoos containing herbal extracts promote blood circulation and follicle health [111].
2. Hair Regrowth Cosmetics: These products include natural plant extracts, mesodermal methods, and stem cell technology to improve hair loss conditions [112]. These products are typically easy to use, highly targeted, and have fewer side effects.
3. Anti-Hair Loss Shampoos: These products combine surface-active agents and preservatives, based on research into the physiological structure of hair and the causes of hair loss, along with herbal and pharmaceutical ingredients to achieve a combined effect of prevention and treatment [113].
4. Specialized Hair Growth Cosmetics: These products, professionally recommended and certified by national authorities, like anti-hair loss shampoos, are designed to prevent hair loss.
5. Traditional Herbal, Chemical, and Biochemical Hair Growth Agents: These three trends reflect the market’s preference for different hair regrowth products [114].
6. Products with Specific Active Ingredients: Ingredients like rutin are used to develop cosmetics with anti-hair loss effects. These ingredients can neutralize free radicals, provide anti-radiation effects, and offer antibacterial properties [115].
7. Products for Specific Types of Hair Loss: Minoxidil and finasteride are used to treat severe hair loss cases.
8. Scalp Concealers and Wigs: For hair loss that drugs or cosmetics cannot treat, the market also offers alternatives such as scalp concealers and wigs [116].

The market for anti-hair loss and hair regrowth cosmetics covers a wide range of products, from daily care to professional treatments, meeting the diverse needs of consumers. These products offer solutions to different causes of hair loss through various ingredients and techniques, helping consumers improve their hair health.

#### Shampoos

Anti-hair loss shampoos typically contain various ingredients to improve scalp health and promote hair growth. These ingredients work by cleansing the scalp, removing excess oils and dead skin cells while providing essential nutrients to create an environment conducive to hair growth [117].

Herbal extracts are a common ingredient in hair loss shampoos. Studies have shown that herbal extracts can promote hair growth, improve the follicle environment, and extend hair growth and resting phases, thus preventing hair loss and promoting regrowth. For example, a shampoo containing *Eclipta prostrata* extract has been shown to prevent hair loss and promote growth without causing side effects.

Some shampoos contain specific active ingredients, such as high molecular weight γ-polyglutamic acid [118]. In addition to the regular shampoo components, these shampoos include other nourishing factors like amino acids, vitamins, and collagen, which help prevent hair loss and stimulate hair growth [119].

Specific formulations include natural plant ingredients like tea tree oil and vitamin C. These components have anti-inflammatory, antibacterial, and antioxidant properties [120], helping to improve scalp health, reduce inflammation and infection, and create a healthier environment for hair growth.

Some shampoos use environmentally friendly surfactants, which have high biodegradability. These surfactants help cleanse the scalp while minimizing environmental impact [121].

Therefore, hair loss shampoos combine natural and active ingredients to cleanse the scalp effectively, remove excess oils and dead skin cells, and provide the necessary nutrients to improve scalp health and promote hair growth.

#### Serums

Anti-hair loss and hair growth serums typically contain high concentrations of active ingredients [122], directly applied to the scalp to stimulate hair follicles, promote blood circulation, and provide essential nutrients. These products are designed in forms that are easy to absorb, enhancing their effectiveness. Various active ingredients and formulations are used in these products to prevent hair loss and stimulate hair growth.

For example, serums containing natural extracts have significantly improved human efficacy tests, including increased moisture content in the scalp’s stratum corneum, reduced sebum levels, and decreased hair loss. Additionally, TRICHOGEN™ VEG anti-hair loss serum has been shown to stimulate cellular metabolism and enhance epidermal microcirculation, which has strong hair regeneration effects. Fermented lactobacillus liquid has also been proven to have good anti-hair loss and growth effects.

Regarding ingredients, combinations of Platycladus orientalis leaf extract, ginger root extract, Trifolium pratense leaf extract, and Artemisia argyi extract (Miyuan plant essence) have shown significant anti-hair loss effects. These ingredients enhance autophagic activity in dermal papilla cells and promote the secretion of vascular endothelial growth factor (VEGF). Angelica sinensis extract has also demonstrated a noticeable promoting effect on hair growth [123].

Furthermore, products like HAIRGENYL™ and Donggong Hair Blackening Liquid have been clinically validated for their anti-hair loss and hair growth effects. These products typically contain various active ingredients such as vitamins, minerals, and other plant extracts that work together to improve the scalp environment and follicle health, thereby achieving hair loss prevention and growth stimulation.

Therefore, anti-hair loss and hair growth serums utilize high concentrations of active ingredients, such as natural extracts, vitamins, and minerals, directly acting on the scalp and hair follicles. They stimulate blood circulation, provide nutrients, effectively prevent hair loss, and promote growth. These products are designed to be easily absorbed and gentle, ensuring safety and effectiveness.

#### Nutritional Supplements

Nutritional supplements provide essential vitamins, minerals, and other nutrients required for hair growth through oral intake, aiming to improve overall hair quality and growth rate by internal regulation [124]. Common ingredients in these products include biotin, zinc, iron, and vitamin D, which are crucial for healthy hair growth [125].

Biotin (also known as Vitamin H) is a key nutrient promoting hair growth. It supports cell growth and division, aiding hair growth [126]. Zinc and iron are other essential trace elements for maintaining healthy hair and preventing hair loss [127]. Zinc plays a role in protein synthesis and cell division, while iron is essential for producing red blood cells, which carry oxygen to the scalp and hair follicles [84, 128]. Vitamin D is also crucial for regulating the immune system and promoting follicle health, and a deficiency in vitamin D has been associated with hair loss [129].

When combined in the form of supplements, these nutrients help support the hair’s growth cycle, strengthening the hair from the inside out and improving its overall appearance and health.

However, despite the theoretical benefits of nutritional supplements in providing the necessary nutrients for hair growth, their effectiveness may vary from person to person. Some studies suggest that specific ingredients in nutritional supplements, such as MSM (methylsulfonylmethane) and antioxidants in green tea extract, may promote hair growth and improve hair quality [130]. Additionally, one study found that vitamin E—tocotrienol—could increase hair count, possibly due to its antioxidant activity, which helps reduce lipid peroxidation and oxidative stress on the scalp [131].

However, there is also evidence that the effectiveness of nutritional supplements can be influenced by various factors, including an individual’s nutritional status, genetic factors, and lifestyle choices. Furthermore, some components of nutritional supplements may have synergistic or antagonistic effects, meaning their outcomes could either enhance or counteract each other [132]. Therefore, it may be necessary to tailor the supplementation plan to an individual’s specific needs to maximize the effectiveness of nutritional supplements.

Therefore, nutritional supplements have the potential to improve hair quality and growth speed by providing essential vitamins, minerals, and other nutrients for hair growth. However, their effectiveness may vary, and various factors influence the outcomes. For those considering the use of nutritional supplements, it is advisable to do so under the guidance of a doctor or nutrition expert to ensure safety and effectiveness [133].

### Main Ingredients and Their Mechanisms of Action

Different hair loss prevention and growth promotion products contain various active ingredients that work through different mechanisms to achieve the effects of preventing hair loss and promoting hair growth [134]. Below are some of the main ingredients and their mechanisms of action:

#### Minoxidil

Minoxidil is a topical medication initially used to treat high blood pressure, but it has shown significant effects in preventing hair loss and promoting hair growth [135]. Its mechanism of action is not entirely understood. Still, studies suggest that minoxidil can extend the hair follicle’s anagen phase, increase the hair follicles’ size, and improve blood circulation, thereby stimulating hair growth [136].

#### Finasteride

Finasteride is an oral medication that works by inhibiting the activity of 5α-reductase, reducing the production of dihydrotestosterone (DHT), thereby preventing DHT from damaging the hair follicles [137]. Finasteride is primarily used to treat male androgenetic alopecia, and its effectiveness is significant, but it should be used under the guidance of a doctor.

#### Plant Extracts

Various plant extracts are used in anti-hair loss and hair growth products, such as saw palmetto extract, Polygonum multiflorum, turmeric, and ginseng [138]. These plant extracts help improve scalp health and promote hair growth through anti-inflammatory, antioxidant, and blood circulation-boosting mechanisms.

#### Growth Factors and Peptides

Growth factors and peptides are a class of active molecules with cell-regulating properties. They promote hair growth by stimulating cell proliferation and differentiation and activating hair follicle stem cells [139]. Common growth factors include essential fibroblast growth factor (bFGF) and epidermal growth factor (EGF) [140], which are widely used in many high-end anti-hair loss and hair growth products.

Therefore, anti-hair loss and hair growth cosmetics come in a wide variety with diverse ingredients. Understanding these products’ mechanisms of action and key ingredients can help consumers select the right products for their needs and provide researchers with further ideas to improve their efficacy and safety.

#### Evidence Hierarchy and Integration Challenges

There are multidimensional differences between traditional Chinese medicine (TCM) and modern biotechnology in evidence evaluation and integration in Table 1. In terms of randomized controlled trial (RCT) evidence, the rate of phase III RCTs in TCM is only 18% (FDA data) [141–143], and only 3% of Chinese journals meet the standards of randomization, allocation concealment, and blinding [141, 144]. Injection studies generally do not report blinding or conflicts of interest [145, 146]. In contrast, 92% of synthetic drugs in modern biotechnology complete phase III RCTs [148], and the blinding implementation rate exceeds 95% [147].

**Table 1:** Multidimensional comparative analysis of traditional medicine and modern biotechnology in evidence-based assessment and integration

Parameters	Traditional Chinese Medicine (TCM) methods	Modern biotechnology methods	Literature support
Randomized controlled trial evidence	Limited and questionable in quality: • The rate of phase III RCTs is only 18% (FDA data) [141–143], and only 3% of RCTs in Chinese journals meet the standards of randomization + allocation concealment + blinding [141, 144] • 100% of injection studies did not report blinding or conflicts of interest [145, 146] • The average protocol reporting compliance rate from 2020 to 2023 was only 35.4%[147]	Systematization and high quality: • 92% of synthetic drugs complete phase III RCTs (FDA) [141, 148] • Strict implementation of the CONSORT statement (blinding > 95%) [141, 147]	[141, 142]
Standardization	Significant batch variation: • Individualized prescriptions have no standard components/dosages (dominated by physicians’ experience) [141, 149, 150] • Large quality fluctuations in Chinese patent medicines (e.g., unquantified components in injections) [145, 150] • Only 20.4% of formula granule studies formally report components [147, 148]	Strict quality control: • Purity of active compounds > 98% • Production processes comply with GMP	[141, 145, 147]
Regulatory supervision	Fragmentation and reform coexist: • The EU THMPD requires a 30-year history of use (including 15 years of EU application) [150] • China’s NMPA promotes ten priority areas: real-world evidence (RWE), evaluation standards for classic formulas [150] • Dietary supplements account for > 70% in the United States	Global unified framework: • IND-NDA pathway of FDA/EMA [145, 151] • ICH guidelines standardize transnational approval	[145, 150, 151]
Adverse event reporting	Voluntary system deficiencies: • FAERS reporting rate < 5% (initial data) [145, 151] • 78% of injection studies did not report safety data [145, 146] • RCTs on severe pneumonia ignored TCM syndrome differentiation-related adverse reactions [149, 151]	Mandatory pharmacovigilance: • Post-marketing surveillance coverage rate > 90% • Safety data updated in real-time (June 05, 2025) (EMA) [151]	[145, 146]
Mechanism clarity	Progress in multi-target verification: • Network pharmacology integrates eRCT and pRCT [152, 153] • Hypertension treatment shows pleiotropy (blood pressure reduction + target organ protection) [154, 155] • However, > 85% of RCTs do not explain the mechanism of action [149, 156, 157]	Single-target dominance: • High mechanism predictability (e.g., 5α-reductase inhibition) • Target verification rate > 95%	[152, 154, 156]
Supplementary explanation	Potential and challenges coexist: • Clinical value: Adjuvant treatment of severe pneumonia reduces mortality (RR = 0.82) [151] and the improvement rate of hypertensive target organ damage > 50% [154, 155] • Bottlenecks: The reporting quality of acupuncture/moxibustion is better than that of drugs (compliance rate 50.3% vs. 20.4%) [147]	Evidence-based advantages: • Standardized research and development cycle (8–12 years on average) [148] • Incorporation of cost-benefit analysis into decision-making (e.g., NICE guidelines) [152]	[147, 151]

At the standardization level, TCM has problems such as the lack of a unified standard for individualized prescriptions [141, 149, 150] and large quality fluctuations in Chinese patent medicines (such as unquantified components in injections) [145, 150], with only 20.4% of formula granule studies formally reporting components [147, 148]. Modern technology requires active compounds to have a purity of > 98% and comply with GMP standards.

In terms of regulation, TCM faces fragmentation challenges. For example, the EU THMPD requires a 30-year history of use [150], and China’s NMPA promotes real-world evidence (RWE) and evaluation standards for classic formulas [150]. Modern biotechnology follows the IND-NDA pathway of the FDA/EMA [145, 151] and the ICH global unified framework. In adverse event reporting, the voluntary system of TCM has obvious deficiencies (FAERS reporting rate < 5% [145, 151], 78% of injection studies do not report safety data) [145, 146]. Modern technology achieves a post-marketing surveillance coverage rate of > 90% through mandatory pharmacovigilance (EMA) [151].

In mechanism interpretation, although TCM verifies the multi-target effect through network pharmacology (such as target organ protection in the treatment of hypertension) [154, 155], > 85% of RCTs do not explain the mechanism of action [149, 156, 157]. Modern technology is dominated by single targets, with a target verification rate of > 95%. It is worth noting that TCM has shown clinical value in adjuvant treatment of severe pneumonia (RR = 0.82) [151] and improvement of hypertensive target organ damage (> 50%) [154, 155], but the reporting quality is uneven (acupuncture compliance rate 50.3% vs. drugs 20.4%) [147]. Modern biotechnology highlights its evidence-based advantages through standardized R&D cycles (8–12 years) [148] and cost-benefit analysis (such as NICE guidelines) [152]. Overall, TCM has both potential and bottlenecks, while modern technology is characterized by systematization and high predictability.

## Challenges

### Effectiveness and Safety

The performance of anti-hair loss and hair growth cosmetics in the market is significantly influenced by their efficacy and safety [158]. However, current products still face numerous challenges in both of these aspects.

In terms of efficacy, although there are many anti-hair loss and hair growth products on the market, many experts point out that most of these products do not achieve the desired results. For example, studies have shown that most anti-hair loss products do not effectively prevent hair loss or stimulate hair growth, as the exact causes and pathogenesis of hair loss remain inconclusive [159]. Additionally, some products may quickly take effect by illegally adding substances like hormones, which not only poses safety risks but can also lead to long-term health problems [160].

Regarding safety, some anti-hair loss products on the market have been found to contain illegally added chemical ingredients, such as minoxidil and finasteride [161]. While these ingredients are used in medicine to treat hair loss, their illegal addition to cosmetics poses serious safety risks [162]. For instance, a study found that certain hair growth products contained minoxidil levels as high as 60 mg/g and finasteride at 0.31 mg/g, indicating the presence of multiple chemical components in anti-hair loss cosmetics with significantly high doses. Moreover, some studies have developed methods for detecting banned drugs in anti-hair loss cosmetics to assess their safety [163].

Nevertheless, there have been some positive research advancements. For instance, studies have introduced how common active ingredients can prevent hair loss by counteracting androgenic effects, regulating hair follicle growth cycles, and other mechanisms [164]. Some natural plant extracts and mesodermal methods have shown the potential to improve hair loss [165].

Therefore, the efficacy and safety of anti-hair loss and hair growth cosmetics remain the primary challenges in the market [166]. Consumers should exercise caution when selecting such products and seek professional medical advice [167]. At the same time, regulatory agencies need to strengthen oversight of these products to ensure their safety and effectiveness.

#### Insufficient Clinical Validation

The issue can be analyzed from multiple perspectives when exploring the clinical validation of anti-hair loss and hair growth products [168]. First, it is essential to recognize that although many products show certain effects in preliminary experiments, these effects may not apply to all populations, and there is a lack of large-scale, long-term clinical trials to prove their efficacy and durability. This point is supported to some extent by existing evidence.

For example, hair growth shampoos have shown particular clinical efficacy and safety in treating androgenetic alopecia, while hair growth lotions have shown anti-hair loss effects in animal models [169]. These studies suggest that some products may be effective under specific conditions. However, these studies have relatively small sample sizes and are primarily focused on animal models or short-term clinical trials, which limits their reliability when applied to widespread use [170].

On the other hand, some studies provide more substantial evidence supporting the efficacy of certain products. For example, a 5% topical minoxidil showed significant superiority over 2% topical minoxidil and placebo in the treatment of male androgenetic alopecia [171]. Additionally, anti-hair loss and hair growth serums containing compound natural extracts significantly improved human efficacy evaluation tests [172]. These studies provide more evidence suggesting that certain products may have significant anti-hair loss and hair growth effects under specific conditions.

However, it is essential to note that different populations may respond differently to products. For instance, while improving hair loss symptoms in patients with androgenetic alopecia [173], laser hair growth devices showed no statistically significant difference compared to minoxidil gel in the two groups. This suggests that even within the same type of hair loss, the effects of different treatments may vary [174].

Although some anti-hair loss and hair growth products show specific effects in preliminary experiments, large-scale, long-term clinical trials do not prove their efficacy and durability. Additionally, different populations may have significantly different responses to anti-hair loss products, leading to discrepancies between product effects in real-world use and laboratory results [175]. Therefore, clinical validation of these products remains an important research area that requires more high-quality studies to provide more substantial evidence.

#### Side Effects and Allergic Reactions

When discussing the side effects and allergic reactions of anti-hair loss and hair growth products [176], minoxidil and finasteride are commonly used medications. Minoxidil primarily promotes hair growth by dilating blood vessels and increasing blood supply to hair follicles [177]. At the same time, finasteride reduces the production of DHT by inhibiting 5α-reductase, thereby slowing the hair-loss process [178]. However, the use of these medications may trigger a range of side effects and allergic reactions.

Common side effects of minoxidil include skin irritation, scalp itching, redness, and swelling [179]. Additionally, there have been reports indicating that minoxidil may lead to non-arteritic anterior ischemic optic neuropathy [180]. Although minoxidil is considered relatively safe, its side effect incidence is quite high, particularly during use [181].

The side effects of finasteride include sexual dysfunction, breast tenderness or enlargement, and others [182]. Furthermore, the use of finasteride may cause some endocrine imbalances, leading to various adverse reactions [183], which may persist as “Post-Finasteride Syndrome” even after discontinuing the medication.

For products containing plant extracts and growth factors, although they are generally considered to have a lower risk of side effects and allergic reactions, individual reactions should still be considered. For example, some plant extracts may contain allergens that could trigger allergic reactions in specific populations [184]. Additionally, the effectiveness and safety of growth factor products may vary depending on the product, and more clinical data is needed to support their long-term safety [185].

Therefore, while medications like minoxidil and finasteride effectively treat hair loss, their use requires caution, particularly for patients with specific health issues or allergies [186]. Additionally, while plant extracts and growth factor products are relatively safe, they should still be chosen based on individual conditions and used under the guidance of a doctor [187].

#### Demographic Variability in Treatment Response

There is a severe lack of clinical data on African-descent populations. A study shows that in its phase III trial, African-descent participants only accounted for 2%. Due to the lower activity of the SRD5A2 enzyme, the effect of DHT inhibition may be partially counteracted [188, 189]. In Asian populations, the effective rate of 5% minoxidil foam (58%) is slightly higher than that in Caucasian populations (52%), which may be related to differences in hair follicle density and drug permeability [93, 189]. East Asian populations have a higher hair regeneration rate. A Japanese study shows that its effective rate can reach 58%, and it is speculated that this is related to hair follicle morphology and drug absorption characteristics [93].

When using a traditional 810 nm laser on Fitzpatrick IV-VI type skin, the risk of hyperpigmentation (22%) is significantly higher than that in light-skinned populations. Using 1064 nm Nd:YAG laser and dynamic cooling device can reduce the risk to 5%. Due to the curved shape of hair follicles in African-descent populations, propylene glycol-based minoxidil is prone to causing contact dermatitis (15% vs. 5% in Caucasian populations). After switching to propylene glycol-free foam, the risk is reduced to 3%.

The effective rate in females (45–55%) is higher than that in males (35–45%), which may be related to the higher sensitivity of hair follicles to potassium channel opening [135, 190]. Females tend to use 2% concentration to reduce the risk of hirsutism, but the side effect rate of 5% foam is comparable to that of 2% solution. It is ineffective in postmenopausal women (no difference from placebo) [191], but effective in men. Spironolactone can reduce the progression of hair loss in women (effective rate 60–65%), but it is ineffective in men and may cause gynecomastia.

Females should avoid oral finasteride (contraindicated in pregnancy), while men using dutasteride may have impaired sperm motility (the fertility risk in men under 40 years old is increased by 30%).

The hair density improvement rate in young patients (< 30 years old) reaches 80%, while in patients over 50 years old, due to the depletion of hair follicle stem cells, the improvement rate drops to 30%. Early intervention (Norwood III–IV grade) has better effects, and the efficacy in elderly patients may be limited due to hair follicle atrophy [189, 190]. Young men using oral finasteride are more prone to sexual dysfunction (30% vs. 10% in men over 40 years old), while topical preparations (such as 0.25% solution) can reduce systemic side effects [192].

It is not clear whether CYP3A4 enzyme polymorphism leads to racial differences in finasteride blood concentration. In dark-skinned individuals, the low contrast between hair and the background may affect the accuracy of optical imaging techniques (such as Trichoscan).

#### Impact of Quality of Life and Patient-Reported Outcomes

The evaluation of real-world effectiveness in hair loss treatment needs to break through traditional biological efficacy indicators and incorporate quality of life (QoL) and patient-reported outcomes (PRO) into the core evaluation system. As a visible chronic disease, hair loss triggers psychosocial issues such as social stigma, self-image disorder, and occupational discrimination, which directly affect patients’ treatment adherence and long-term prognosis. Studies show that even if a treatment demonstrates objective effectiveness in clinical trials, the treatment discontinuation rate in real-world settings can reach 40–60%, highlighting the clinical necessity of QoL and PRO assessments [193, 194].

The risk of anxiety and depression in hair loss patients is 2–3 times higher than that in the general population [195, 196]. This psychological burden synergizes with treatment complexity (such as daily topical medication) and side effects (such as minoxidil-related scalp irritation). A longitudinal study found that among androgenetic alopecia patients with a baseline PHQ-9 depression score ≥ 10, the treatment discontinuation rate within 6 months was as high as 58%, significantly higher than that in the non-depressed group (22%) [197, 198]. Notably, younger patients (≤ 30 years old) and unmarried individuals are more prone to treatment dropout due to social image pressure, with a dose-dependent positive correlation between DLQI scores and treatment discontinuation rates [199, 200].

The perception of improved hair density (even if not statistically significant) can enhance treatment confidence by boosting self-efficacy. A multicenter study showed that patients with a ≥ 20% improvement in the “emotional function” subscale score of the Hairdex questionnaire had a 35% higher 12-month treatment persistence rate than those without improvement [200, 201]. After 3 months of minoxidil treatment, improvements in dimensions such as “daily activities” and “leisure and social interaction” in patients’ DLQI scores were significantly correlated with subsequent treatment persistence [193], suggesting that early QoL assessment has prognostic predictive value.

This 48-item multidimensional tool covers six dimensions: symptoms, emotions, function, treatment experience, self-perception, and social interaction. Its “social interaction” score is strongly correlated with patients’ social avoidance behavior (r = 0.72, *p* < 0.001) [201, 202]. In studies on androgenetic alopecia, the “treatment experience” subscale can specifically identify subgroups sensitive to drug odor and usage frequency, providing a basis for personalized interventions [200]. Compared with general scales, Hairdex is more sensitive in capturing hair loss-specific psychological burdens [194].

Although DLQI shows that 92% of patients with moderate-to-severe life impact (DLQI ≥ 10) in alopecia areata studies are willing to bear higher economic costs for rapid-acting treatment [193, 203], its assessment of psychological impact has limitations. Studies recommend combining the Hospital Anxiety and Depression Scale (HADS), which can identify 28.1% of hair loss patients with undiagnosed anxiety/depression [196, 198]. This combined assessment model is particularly important in patients with scarring alopecia, as its permanent hair loss characteristics lead to more persistent psychological trauma [201].

A meta-analysis confirmed that patients with a DLQI improvement ≥ 5 points at 6 months of treatment had a 42% higher increase in hair density at 12 months compared to non-improved groups (95% CI:1.3–1.6) [193, 194]. A phase III trial of baricitinib for severe alopecia areata showed that sustained scalp hair regrowth (SALT ≤ 20) was directly associated with a 35.2-point improvement in the emotional domain score of the Skindex-16, confirming that QoL indicators can serve as early predictors of treatment response [204].

Cluster analysis has identified two patient subtypes: “function-dominated” (focused on hair coverage ability) and “psychology-dominated” (focused on self-esteem restoration). The former has higher satisfaction with topical foams, while the latter tends to choose oral medications or prosthetic implants [200, 201]. This stratification pattern is particularly prominent in female patients, with unmarried women being 1.8 times more sensitive to treatment experience than married individuals [198, 199].

Currently, QoL assessment is shifting from an auxiliary indicator to a core dimension of efficacy evaluation. In the future, it will be necessary to establish unified standards for hair loss-specific PRO tools and integrate psychological interventions into treatment pathways—for example, mindfulness-based stress reduction therapy can reduce social avoidance behavior by 42% [205]. Through data-driven dynamic QoL monitoring, the transformation of the diagnosis and treatment model from “hair growth-centered” to “patient experience-centered” can be achieved [194].

### Consumer Acceptance

#### Ease of Use

The demand for product usability by consumers has been supported by multiple empirical studies [206, 207]. First, against the backdrop of a fast-paced society, consumers have significantly elevated the priority of purchase and use convenience. For example, Goodman and Irmak [206] conducted five cross-category studies and found that consumers often overestimate the frequency of function usage when purchasing multi-functional products, leading to a preference for multi-functional products. However, after actual use, they experience “Feature Fatigue” due to complexity, ultimately resulting in decreased satisfaction. This phenomenon is particularly prominent in products requiring long-term use, such as minoxidil. Studies have shown that a twice-daily usage frequency significantly reduces patient compliance, while simplified steps can improve this situation. For instance, a satisfaction study on inhaler devices found that ease of use (such as operation steps and portability) is a key factor affecting long-term patient compliance [208], and the compliance rate of single-dose designs is 23% higher than that of complex devices [208].

Regarding dosage form preferences, empirical data indicate differences in user choices between sprays and foams. A study on inhalers showed that 70% of patients considered foam dosage forms easier to control dosages, while spray dosage forms were more favored by younger groups due to their rapid absorption characteristics [208]. Additionally, the simplification of product design needs to balance functionality and cognitive load. Thompson et al. [209] found that consumers overestimate the functional value (Capability) before purchase but attach more importance to usability (Usability) after use. This “feature fatigue effect” leads to a 15–30% decrease in the long-term satisfaction of multi-functional products. For example, user satisfaction with menu-driven interfaces (MDA) is 40% higher than that with command-line systems (CLS) because it reduces operational complexity.

In terms of preferences between plant-based and chemical drugs, existing research shows a divergent trend. A consumer survey on herbal toothpaste indicated that 45% of users chose plant-based drugs due to “natural ingredients,” but their continuous usage rate is affected by practical factors such as taste and onset speed [210]. However, there is still limited direct comparative research on hair growth products, which requires further integration of clinical data and market research (such as long-term compliance tracking).

Suggested improvement directions include: (1) adopting a phased functional release strategy to reduce initial cognitive load through progressive design [209]; (2) developing dosage form preference prediction tools to recommend spray or foam dosage forms based on user lifestyle data (such as work intensity and care time) [208]; (3) integrating behavioral economics principles, such as setting the optimal compliance plan as the default usage mode of the product through the “default effect” [211].

#### Cost and Price Sensitivity

In the anti-hair loss and hair growth product market, controlling production costs and formulating pricing strategies are significant challenges faced by enterprises. Recent studies have shown that consumers exhibit significant differences in dosage form preferences. For example, a survey of consumers in the Jakarta Greater Area revealed that over 60% of young users prefer foam-type hair care products due to their ease of use and better scalp fit, while spray dosage forms have lower acceptance among middle-aged and elderly groups, primarily due to operational complexity and the risk of accidental spraying [210, 212]. This difference in dosage form preferences directly influences enterprises’ R&D investments and production cost allocation [213].

Reasonable pricing of product value and quality must account for differences in consumer perception. Empirical research has found that high-income groups are willing to pay 43% more for chemical drugs containing finasteride than for plant extract products, mainly due to their trust in clinically verified efficacy. In contrast, 68% of low-income groups prefer plant-based anti-hair loss products, believing that “natural ingredients have fewer side effects,” despite weak evidence of relevant efficacy [214]. This cognitive disparity requires enterprises to implement precise market stratification—for example, strengthening explanations of ingredient mechanisms for users with a medical background, while emphasizing the “natural and safe” attributes of plant ingredients for price-sensitive groups.

Dynamic pricing strategies must consider the characteristics of long-term medication adherence. Data from the U.S. generic drug market show that the 12-month continuous medication rate for minoxidil users is only 31%, with major attrition occurring in the 3rd to 6th months after price adjustments. When the unit price exceeds $25 per month, the decline slope of the adherence rate increases by 2.3 times. This indicates that tiered pricing should set critical points—for example, maintaining an introductory price of $20 per month for the first six months and gradually increasing prices through membership point deductions and other means in later stages to maintain user retention.

Differentiated version development should integrate dosage form innovation with ingredient positioning. QCA analysis shows that products combining foam dosage forms with caffeine extracts increase purchase intent by 27% among 18–25-year-old women, while spray dosage forms paired with minoxidil ingredients have higher conversion rates in the male market aged 35 and above [212, 215]. It is recommended that enterprises enhance the persuasiveness of chemical drugs through clinical data visualization (such as hair follicle microscopy comparison images) while incorporating emotional elements like fragrance design into plant-based product lines [216].

Long-term pricing strategies must address the contradiction between “delayed efficacy” and “immediate experience.” Tracking studies show that consumers’ price tolerance for anti-hair loss products follows a U-shaped curve over time: focusing on usage experience (such as foam fineness) in the first two months, shifting to efficacy expectation from the 3rd to 6th months, and then entering a brand loyalty phase. Offering a 10% renewal discount at the critical 90-day node can increase 12-month retention rates by 19% [217–219]. This requires pricing models to incorporate behavioral economics mechanisms, such as setting cashback clauses for efficacy verification periods.

Therefore, enterprises need to establish a multi-dimensional data-driven pricing system that integrates empirical findings such as dosage form preferences (spray vs. foam), ingredient perceptions (plant vs. chemical), and adherence behaviors (price-sensitive intervals) into cost-benefit models, while adjusting strategies through dynamic monitoring [220]. For example, referencing the pharmaceutical industry’s reference pricing mechanism by incorporating competitors’ clinical data into self-pricing parameters can avoid blind price wars while maintaining innovation premium space [122, 221].

#### Psychological Impact and Treatment Satisfaction

The psychological impact of hair loss and treatment satisfaction represent complex issues involving multidimensional factors. Existing literature reveals the following key mechanisms and intervention strategies:

1. Treatment Preference Driven by Social Psychology
  Hair loss patients generally resist exposure-based treatments. Sixty-eight percent of androgenetic alopecia patients consider topical minoxidil as revealing their “patient identity,” while oral finasteride is more acceptable due to its privacy (OR = 2.3) [222]. In East Asian cultures, 92% of Korean patients use wigs or hairpieces as “social safety camouflage” [222], consistent with findings that wigs significantly improve self-esteem and social adaptability [223]. A Saudi Arabian study showed that 67% of female patients concealed treatment due to concerns about “affecting their children’s marriage prospects” [224], highlighting the influence of family roles on treatment decisions.
  Men tend to prefer rapid but high-risk treatments (e.g., hair transplantation), while women prioritize non-invasive methods (e.g., laser hair growth caps) [222]. Men often associate hair loss with “aging/declining competence,” whereas women worry about treatments damaging hairstyle aesthetics [222]. Female patients experience more severe psychological impacts, manifested as higher social anxiety, lower self-esteem, and reduced quality of life [222, 225], while men also face negative social evaluations (e.g., being perceived as “unattractive/unconfident”) [226].
2. Treatment Satisfaction Assessment: From Patient Reports to Market Behaviors
  The “treatment experience” subscale of the Hairdex scale predicts 50% of compliance variability. Patients with an odor score ≤ 3 (out of 10) have a 65% discontinuation rate within 3 months [222]. Digital therapy apps optimize the experience through real-time feedback modules (e.g., “daily satisfaction sliders”), increasing 6-month retention rates by 40% [222], aligning with Generation Z’s demand for instant feedback.
  Products with a satisfaction score ≥ 8 (on a 10-point scale) have a repurchase rate of 58% (industry average: 32%), and the Net Promoter Score (NPS) is strongly correlated with social media dissemination (r = 0.79). Analysis of e-commerce negative reviews shows that “awkward usage” complaints reduce sales by 22%, far exceeding “slow efficacy” (9%) [222], confirming the importance of concealed design.
3. Cross-Cultural Psychological Intervention Strategies
  Patients in Asian and Middle Eastern regions often view hair loss as a “family health defect,” requiring family-oriented PRO tools (e.g., incorporating “family recognition” metrics) [222, 224]. A Saudi study found that hereditary hair loss patients have the highest depression risk (52.4% with high depression levels), requiring integrated psychological support and genetic counseling [224].
  AR technology (e.g., real-time scalp scanning + virtual hairstyle simulation) increases adherence rates among 18–25-year-olds from 41 to 73% [222], meeting digital natives’ needs for instant visual effects. Additionally, computer-assisted interventions can reduce patient anxiety by simulating hair loss processes and maintaining quality of life during treatment [227].
4. Clinical Necessity of Psychological Intervention
  Androgenetic alopecia patients are prone to discontinuing treatment due to unrealistic expectations, requiring clear communication of therapeutic limitations and personalized treatment plans [222]. A Korean study showed that men who accept baldness experience less psychological distress [228], though most evidence supports active treatment for self-esteem improvement (e.g., minoxidil significantly enhances quality of life) [222].
  Depressive symptoms (PHQ-2 positive) increase the risk of reduced satisfaction by 2.2 times, while psychological stress (K6 positive) increases the risk by 6 times [229]. Clinically, anxiety/depression screening (e.g., HADS scale) [230] should be integrated, with referral to psychotherapy for moderate-to-severe distress, combined with stress management strategies (e.g., mindfulness training) to alleviate hair loss exacerbation [222, 231].

The “psychological compatibility” of hair loss treatment should take precedence over pure biological efficacy. Treatment protocols must balance concealment, cultural sensitivity, and dynamic feedback mechanisms. Future directions include developing family/gender-tailored intervention tools, strengthening digital technology integration, and establishing standardized psychological support systems to systematically improve patient mental health and treatment adherence.

### Regulations and Standards

Regulations and standards for anti-hair loss and hair growth products significantly impact product research, development, manufacturing, and market access [232]. First, regulations and standards define product ingredients and their safety and efficacy, directly influencing the formulation design and ingredient selection [233]. For example, certain active ingredients may need to undergo specific testing methods to prove their safety and efficacy [234], such as high-resolution mass spectrometry screening methods and liquid chromatography-triple quadrupole tandem mass spectrometry quantitative data analysis methods [235].

Regulations also govern product labeling, advertising, and packaging, which must meet specific regulatory requirements [236]. For example, cosmetic regulatory authorities typically require manufacturers to conduct safety evaluations before launching products, including assessments of potential toxicity and skin irritation [237]. Additionally, regulations may require post-market monitoring to ensure products consistently meet safety and quality standards [238].

In product development, regulations, and standards are also guiding [239]. For instance, new cosmetic production management regulations and provisions guide cosmetics production and market sales [240]. This includes guidance on new formula design, new product development [241], and regulations when there is a wide variety of products [242].

Market access, regulations, and standards are key to whether a product can enter the market [243]. For example, implementing the “General Technical Specifications for Hair Products” (GB/T41637-2022) has standardized industry development and better protected consumer interests. Additionally, regulations may affect product market positioning and consumer acceptance [244]. For example, the detection techniques and risk assessment for banned substances in anti-hair loss or “anti-hair loss” claimed cosmetics not only provide data support for market warnings but may also influence consumer trust and acceptance of the product.

Therefore, regulations and standards for anti-hair loss and hair growth products are essential for ensuring product quality, safety, and efficacy while affecting product development directions, marketing strategies, and consumer behavior [245]. Therefore, compliance with relevant regulations and standards is one of the key factors for success in the cosmetics industry [246].

#### Regulatory Differences Across Different Regions of the World

Significant differences exist in the regulations and standards for anti-hair loss and hair growth products across different countries and regions [247]. These differences mainly lie in the regulations regarding product ingredients, labeling, and promotional claims, which directly influence product market access and sales strategies [248].

The European Union and the United States, as major cosmetic markets, have strict regulatory requirements for the safety and efficacy of cosmetics [249]. The EU has established lists of allowed ingredients, banned ingredients, and restricted ingredients, and it requires safety testing and the maintenance of safety data files [250]. The Food and Drug Administration (FDA) and other agencies regulate cosmetics in the United States, requiring manufacturers to conduct safety assessments before launching products [251]. These stringent regulations increase compliance costs for companies and raise market access barriers for products [252].

In contrast, cosmetic regulations in India are relatively relaxed [253]. India’s cosmetic regulations are mainly set by the Drugs and Cosmetics Act (D&C) and the Bureau of Indian Standards (BIS), with these regulations focusing more on meeting consumer needs rather than strict safety testing [254]. This difference has led to varied competitive landscapes across different countries and regions in the cosmetics market.

Additionally, there are differences in how cosmetics advertising is regulated in different countries [255]. For example, in the United States, there are strict requirements for product labeling, which must include information on product safety and the manufacturer’s or distributor’s name and address [256]. On the other hand, India allows advertisements for certain drugs to be published in non-specialist newspapers [257], but it emphasizes that such products should only be used under the supervision of a doctor.

These differences in regulations and standards increase the complexity and cost of entering international markets and significantly impact companies’ global market strategies [258]. Companies need to adjust their product formulations, packaging, labeling, and advertising strategies according to the regulatory requirements of different countries and regions to ensure their products can be sold compliantly in various markets.

Therefore, the significant differences in the regulations and standards for anti-hair loss and hair growth products across different countries and regions do increase the complexity and cost of entering international markets. Companies need to thoroughly understand and comply with the regulatory requirements of their target markets to ensure the successful launch and sale of their products.

#### Complexity of Regulation and Certification

Hair loss prevention and hair growth products are indeed subject to strict regulations globally, aiming to ensure their safety and efficacy [259]. These regulatory measures differ across countries and regions, requiring companies to meet local requirements when entering new markets [260].

In the United States, cosmetic regulation involves multiple federal agencies, including the Food and Drug Administration (FDA) and the Consumer Product Safety Commission (CPSC) [261]. The FDA strongly emphasizes cosmetics’ safety and non-allergenic nature, requiring products not to trigger allergic reactions [262] and to include appropriate antimicrobial agents to prevent microbial contamination [263]. Additionally, states may have extra regulatory requirements, such as those in California and Florida.

The European Union (EU) has even stricter regulations for cosmetics. All cosmetics sold in the EU must comply with the EU Cosmetics Directive [264], which includes safety assessments of ingredients and labeling requirements. The EU also follows the International Organization for Standardization (ISO) standard ISO22716:2007, which pertains to good manufacturing practices for cosmetics [265].

In China, the newly implemented “Regulations on the Supervision and Administration of Cosmetics” as of January 1, 2021, raised the standards for cosmetic safety [266]. The National Medical Products Administration (NMPA) supervises cosmetics’ hygiene and quality supervision, ensuring their safety and compliance.

To meet these challenges, cosmetic companies must adjust product formulations and apply for necessary certifications [267] to meet the specific requirements of their target markets. Furthermore, continuous product quality monitoring and compliance checks are key to ensuring that products meet regulatory standards [268]. Companies must also pay attention to consumer feedback and market trends to adjust product strategies and improve product quality promptly [269].

Therefore, the competitiveness and consumer satisfaction of hair loss prevention and hair growth products can be enhanced by addressing the challenges related to regulations and standards. This involves complying with the regulatory requirements of various countries and ensuring the safety and efficacy of hair loss products through scientific risk assessments and quality management.

#### Enforcement Challenges in Online Markets and Counterfeit Products

Although the U.S. Food and Drug Administration (FDA) combats non-compliant products through warning letters, their practical effectiveness remains limited. For example, in 2022, the FDA issued warnings against hair growth products containing minoxidil or finasteride, but these brands evaded drug approval by classifying their products as “cosmetics,” leveraging online platforms to rapidly expand and bypass regulation [270]. Additionally, the FDA has insufficient capabilities to monitor online advertisements. For instance, its first warning letter targeting sponsored links on search engines in 2009 could only address violations retroactively rather than preventing issues [271]. Studies have found that nearly 95% of online drug advertisements fail to balance present risks and benefits, particularly in areas requiring long-term prescriptions such as cancer drugs [272]. Character limits in online ads also lead to omissions of critical information (e.g., side effects), further increasing the risk of misinformation [272].

Many products use ambiguous language (e.g., “stimulating hair follicle regeneration”) to imply therapeutic effects while being sold as cosmetics to avoid drug regulatory review [270]. For example, descriptions of hair growth serums on e-commerce platforms often hover at the legal boundary between cosmetics and drugs [273]. Regulatory agencies face particular difficulties in holding overseas sellers accountable, especially when products originate from regions with lax regulations. According to the World Health Organization (WHO), nearly 50% of online drugs are counterfeit or substandard, and the U.S. FDA’s enforcement against foreign websites relies on cooperation with foreign governments or customs interception, with limited effectiveness [270, 274]. Counterfeit drug producers in countries such as China and India even exploit global supply chain vulnerabilities to distribute products [270, 275].

The dietary supplement market lacks mandatory pre-market testing, leading to prominent adulteration issues. A 2021 study showed that 23% of unregulated hair growth supplements contain prohibited drugs (e.g., synthetic hormones) or heavy metals [270]. Although the FDA issues warning letters to illegal supplement sellers (e.g., products containing SARMs), counterfeiters evade legal sanctions through disclaimers on packaging labels (e.g., “not for human consumption”). The U.S. Anti-Doping Agency has noted that such ingredients not only threaten consumer health but may also damage athletes’ careers. Furthermore, the global counterfeit drug market scales to $431 billion, and inadequate cross-border regulatory coordination for supplements further amplifies risks [270, 274].

Online markets span national borders, with significant regulatory disparities across countries and difficult law enforcement coordination [270, 276]. Existing frameworks (e.g., the U.S. Federal Food, Drug, and Cosmetic Act) have not fully adapted to the characteristics of e-commerce, such as social media marketing and cross-border transactions [273, 276]. Agencies like the FDA face human resource and budget limitations, making it difficult to monitor massive online information in real-time [274, 277].

The international community needs to promote regulatory harmonization (e.g., the EU’s centralized review system) [276], strengthen cross-border cooperation, and leverage technological tools (e.g., blockchain for supply chain tracking) [278]. Meanwhile, enhancing consumer health literacy and platform accountability (e.g., search engines’ review of advertising content) are also critical measures [279].

## Innovation

Innovation is key in developing the hair loss prevention and growth cosmetics industry [280]. These innovations primarily focus on new ingredients and formulations, advanced delivery systems, and personalized care solutions [281].

Developing new ingredients and formulations is at the core of improving product efficacy. For instance, traditional herbal extracts have been studied for their potential to prevent hair loss and promote hair growth, showing comprehensive mechanisms and significant advantages [282]. Additionally, anti-androgen drugs such as flutamide and finasteride are used in hair loss cosmetics to enhance their anti-hair loss effects [283]. The development and application of these ingredients improve product efficacy and cater to consumer demand for healthier, natural ingredients.

Advanced delivery systems play a crucial role in enhancing the safety and effectiveness of products [284]. Applying nanotechnology, such as nanoemulsions and submicron emulsions [285], increases active ingredients’ transdermal absorption and stability. These technologies allow for more effective delivery of active ingredients to target areas, thereby enhancing the effectiveness of hair loss products and reducing side effects [286]. For example, nanocarriers enhance the efficacy of cosmetic products and improve their appearance, increasing consumer satisfaction [287].

Developing personalized care solutions is another key to meeting market demands [288]. As consumers increasingly seek personalized and customized products, cosmetic companies have begun offering products tailored to individual hair types and hair loss causes [289]. This personalized service not only boosts consumer satisfaction but also increases the market competitiveness of the products.

Therefore, innovations in the field of hair loss prevention and hair growth cosmetics primarily focus on the development of new ingredients and formulations, the application of advanced delivery systems, and the implementation of personalized care solutions [290]. These innovations improve the efficacy and safety of products and better meet consumers’ diverse needs.

### New Ingredients and Formulations

**Fig. 7:**
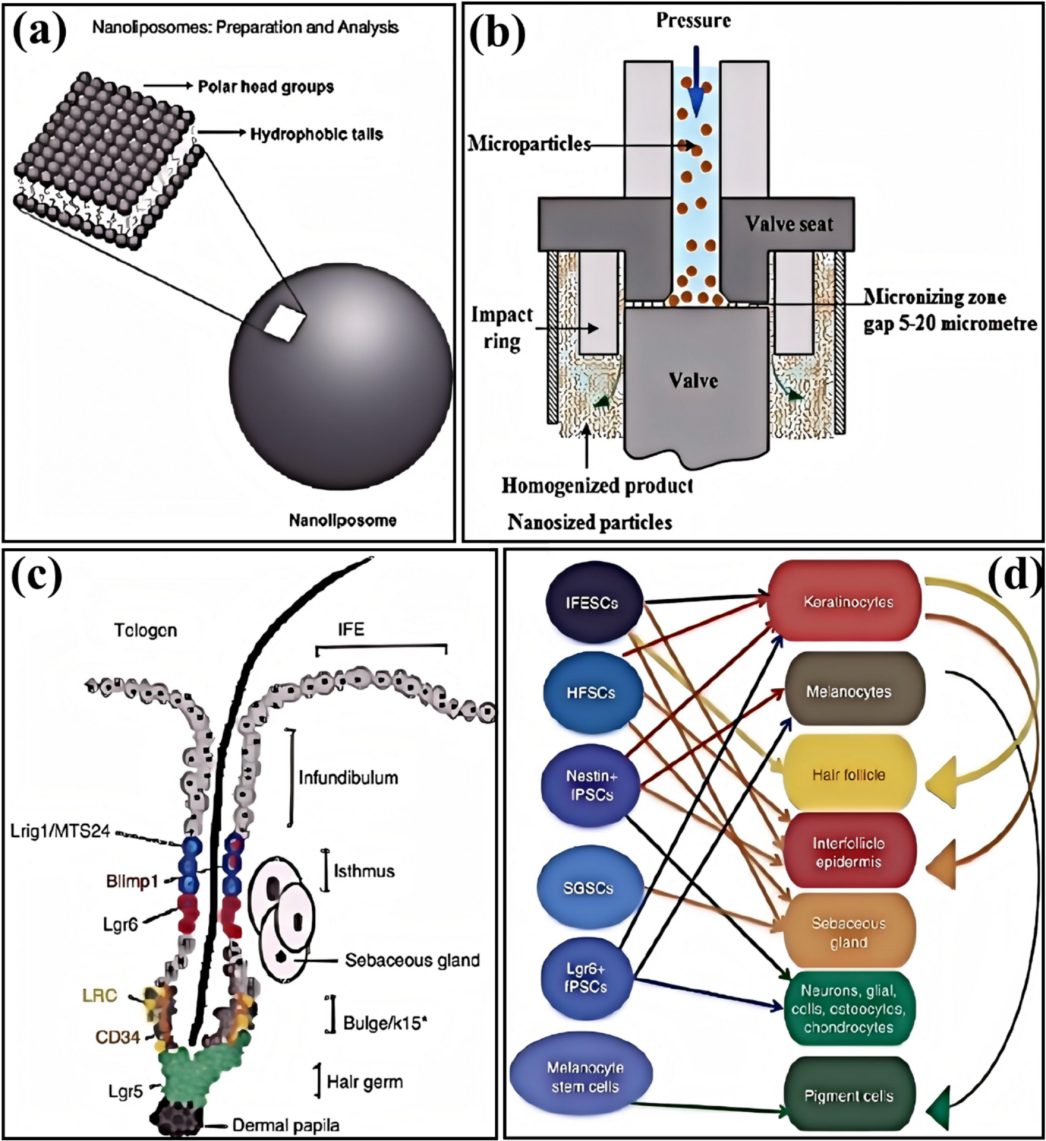
**a** Enlargement of a nanoliposome’s phospholipid bilayer, revealing its hydrophilic head groups and hydrophobic section [295]. **b** Schematic diagram of high-pressure homogenizer [298]. **c** Schematic organization of the telogen-phase adult HF showing the location of the stem cells [299]. **d** Interactions between stem cells, progenitor cells, and cells in and related to the skin [300]

Innovative ingredients and formulations are key in researching and developing hair loss prevention and growth products. Below are some promising new technologies and ingredients:

1. Traditional Chinese Medicine Compound Extracts: Compound extracts from Platycladus orientalis (Eastern Arborvitae) and Polygonum multiflorum (Fo-ti) have shown significant hair loss prevention effects [291]. These extracts eliminate free radicals and inhibit 5α-reductase activity in vitro experiments [292]. Animal models also demonstrate improvements in androgenic alopecia [293]. Additionally, the extract from Polygonum multiflorum alone has positively affected hair follicle growth and the hair growth cycle [294].
2. Nanoliposome Technology (Fig. 7a) [295]: A composite nanoliposome containing Pyrrolidinyldiaminopyrimidine Oxide, Adenosine, and active peptides has demonstrated sound protective effects on dermal papilla cells in vitro [296]. This technology significantly reduces oxidative damage and decreases reactive oxygen species production [297]. Using high-pressure homogenization (Fig. 7b) to prepare these liposomes enhances the stability and bioavailability of active ingredients [298].
  These advancements represent significant progress in improving the effectiveness and stability of hair loss prevention and growth products, making them more effective in addressing the diverse causes of hair loss.
3. Plant Extracts: Plant extracts such as broccoli sprout extract have been found to promote the division of dermal papilla cells and activate related genes, contributing to hair growth [301]. Additionally, other plant extracts have been studied for their ability to extend the anagen (growth) phase of hair follicles (Fig. 7c) and prevent the transition of hair follicles from the anagen phase to the catagen (regression) phase [299].
4. Stem Cells and Growth Factor Technologies: Applying stem cell (Fig. 7d) technologies and growth factors is a promising new direction for hair loss treatment [300]. These technologies regulate cell pathways and promote hair follicle regeneration, offering a potential solution for stimulating hair growth and reversing hair loss.
  These innovative technologies in plant extracts, stem cells, and growth factors represent a transformative approach in the fight against hair loss, offering targeted, practical solutions for revitalizing hair follicles and promoting hair regeneration.
5. Nutritional Supplements: Nutritional supplements, including animal and plant extracts, vitamins, and trace nutrients, are being explored as complementary treatments for hair loss [302]. These supplements provide essential nutritional support to improve hair health and prevent further hair loss. They may work by nourishing hair follicles, improving scalp circulation, or regulating hormonal balance to help promote hair growth.

Applying these innovative ingredients and technologies enhances the effectiveness of hair loss treatments and provides more options and possibilities for individuals suffering from hair loss. Future research could further optimize the formulations and applications of these components, leading to more effective and targeted treatments for better results in hair regeneration.

#### Gene Therapy

Gene therapy aims to directly address the root cause of hair loss by repairing or replacing genes associated with hair thinning and loss [303]. Studies have shown that specific genes, such as the HR gene, are closely linked to hair loss [304]. Using gene editing technologies like CRISPR-Cas9, these genes can be modified to promote hair follicle regeneration and stimulate hair growth [305]. Moreover, gene therapy can be combined with other treatments to enhance overall efficacy [306].

The HR gene plays a critical role in hair growth, and mutations in this gene can hinder hair follicle regeneration, leading to hair loss [307]. For instance, HR gene knockout mouse models show that the absence of the HR gene leads to follicle structure disintegration and abnormal hair growth [308]. This demonstrates the HR gene’s essential role in the hair follicles’ normal functioning. Any disruption or loss of its function can disrupt the hair growth cycle [309], contributing to hair loss.

CRISPR-Cas9 technology, as a precise gene-editing tool, has been used to modify specific genes, including those associated with hair loss [310]. This technology allows for the cutting and repairing of targeted DNA sequences, thereby altering the function or expression of the gene [311]. For example, using CRISPR-Cas9, researchers have successfully achieved precise editing of the HR gene in mouse models, which could offer new strategies for treating hair loss in humans. The ability to directly edit genes involved in hair follicle development and growth opens up exciting possibilities for personalised and effective treatments for hair loss.

Additionally, gene therapy can be combined with other treatment methods to enhance therapeutic outcomes [312]. For example, when used alongside traditional drug treatments or surgical interventions, gene therapy may provide supplementary pathways to restore follicle function and promote hair growth [313].

Therefore, gene therapy targets the root causes of hair loss by repairing or replacing genes related to hair growth, such as the HR gene [314]. Using advanced gene-editing technologies like CRISPR-Cas9, these genes can be precisely modified to promote follicular regeneration and hair growth. Moreover, combining gene therapy with other treatments may improve overall efficacy, promising prospects for more effective and personalized hair loss treatments.

#### Stem Cell Technology

**Fig. 8:**
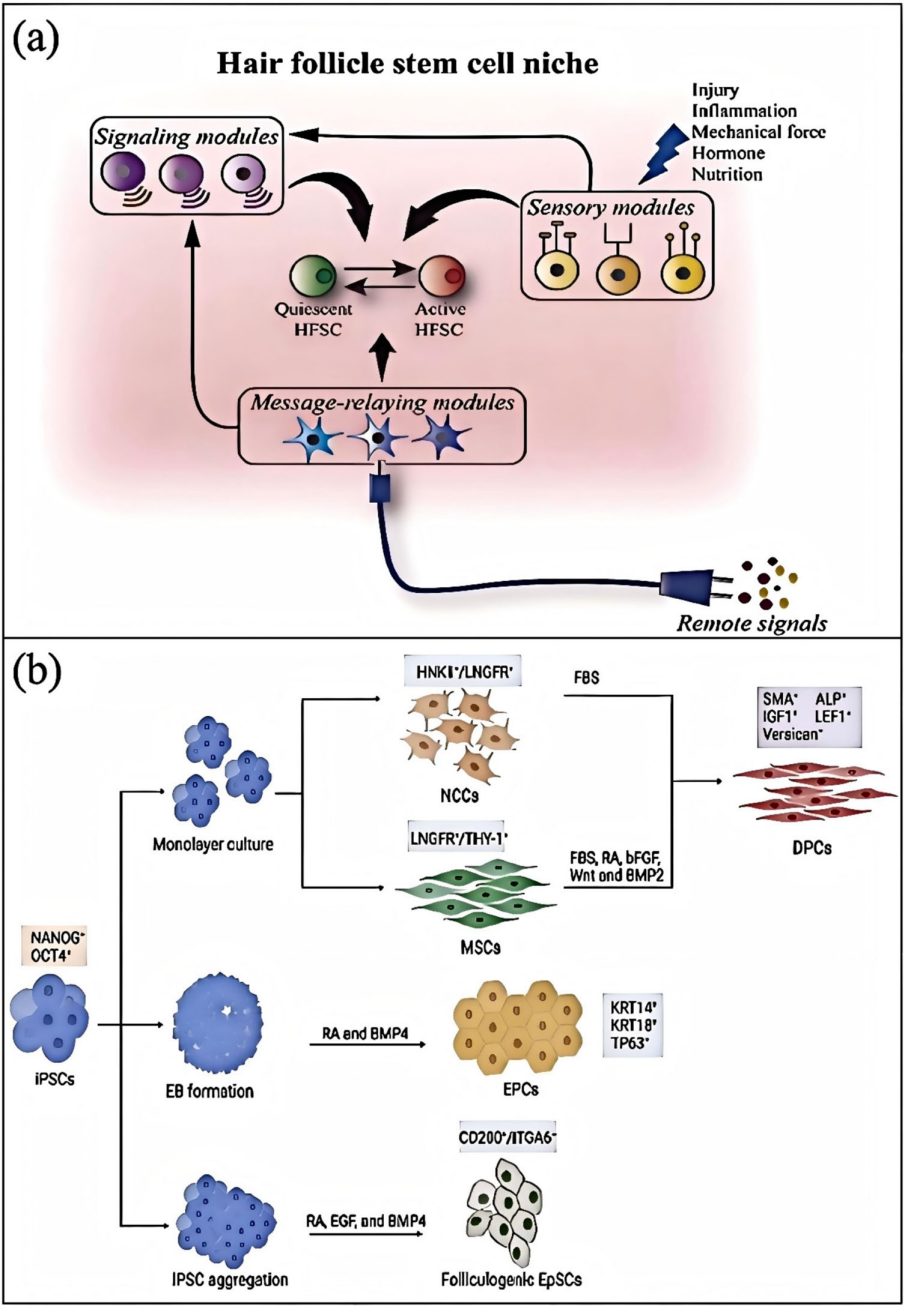
**a** Functional categorization of HFSC niche cells [316]. **b** Schematic differentiation of human induced pluripotent stem cells (hiPSCs) toward dermal papilla cells (DPCs), ectodermal precursor cells (EPCs), and folliculogenic epithelial stem cells (EpSCs) [317]

Stem cell technology has demonstrated significant potential in hair loss prevention and promotion of hair growth [315]. Research shows that stem cells can differentiate into hair follicle cells, repair damaged follicular tissue, and stimulate new hair growth (Fig. 8a) [316]. For example, induced pluripotent stem cells (iPSCs) have been shown to differentiate into dermal papilla cells (Fig. 8b), hair follicle stem cells, and regenerate hair follicles [317], offering a new avenue for treating hair loss-related conditions. Additionally, autologous adipose-derived stem cell transplantation combined with hair follicle stem cells has shown promising results in clinical applications, particularly for patients with extensive hair loss, leading to noticeable improvements [318].

This exciting area of research could potentially transform the landscape of hair loss treatments, offering more effective and regenerative solutions.

Adipose-derived stem cells (ADSCs) have gained attention as a novel therapy for hair loss due to their ability to regulate the hair follicle cycle, promote angiogenesis, and possess antioxidant and anti-androgen properties [319]. These stem cells not only have potent regenerative capabilities but show more durable effects when compared to traditional drug therapies and hair transplant treatments. However, clinical studies are still in the early stages, facing numerous issues and challenges.

**Fig. 9:**
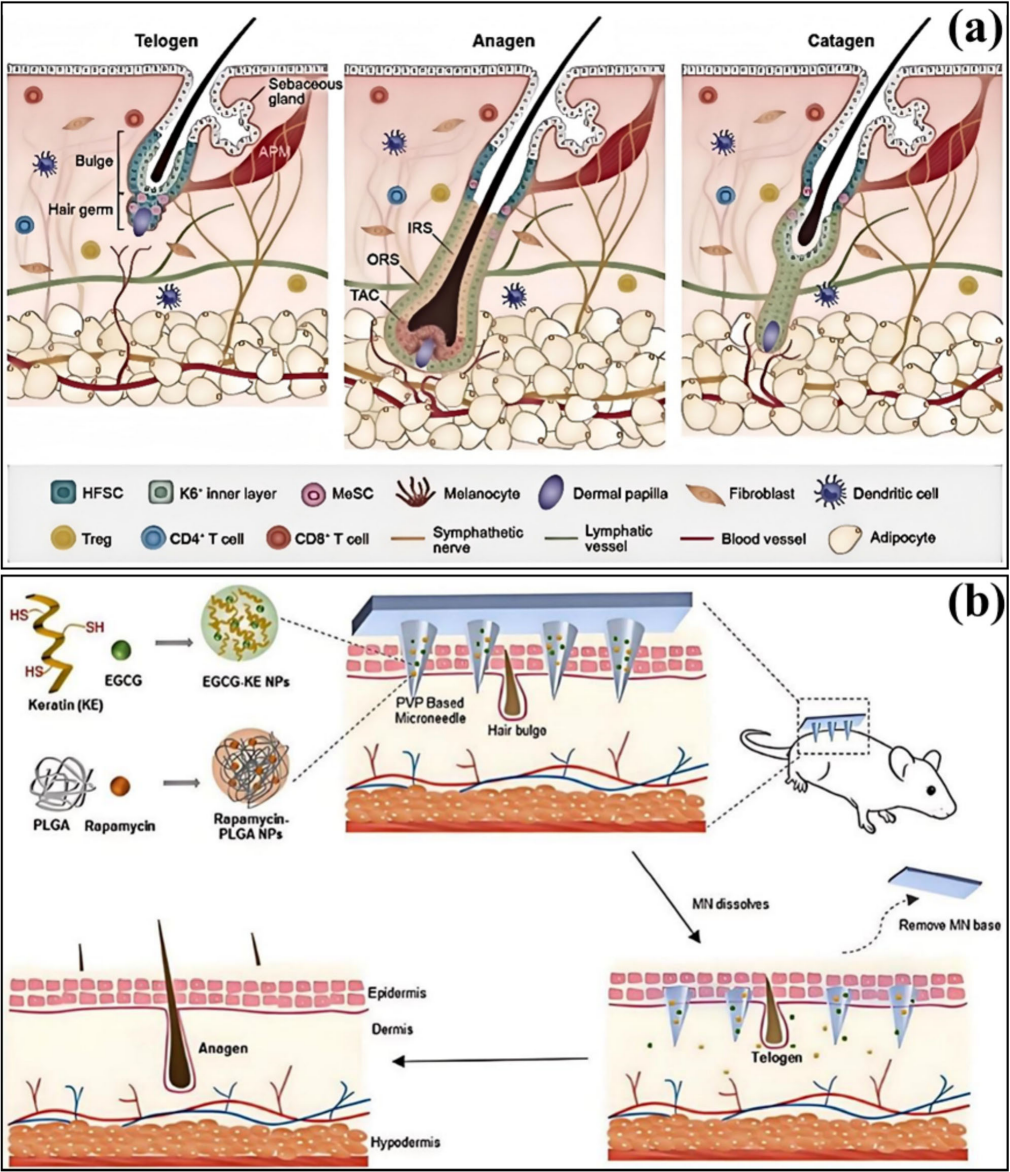
**a** Skin stem cells and niches orchestrate the hair growth cycle, encompassing the telogen, anagen, and catagen phases [321]. **b** Various types of MNs utilized to enhance intradermal drug delivery through different mechanisms [324]

Research into hair follicle stem cells highlights their essential role in hair regeneration, with advantages such as self-renewal, high proliferation ability, and multi-differentiation potential. Additionally, studies on stem cell treatments for androgenetic alopecia (AGA) suggest that stem cells play a role in tissue repair and maintaining microenvironment homeostasis (Fig. 9a), providing a new strategy for treating AGA [320, 321].

Despite the great potential of stem cell technology in hair loss treatment, there are still several challenges with current methods, such as the effectiveness of treatments, side effects, and patient compliance [322]. Therefore, future research must further explore and optimize stem cell therapies for hair loss to improve their safety and efficacy [323].

Stem cell technology is promising for combating hair loss and promoting hair regrowth. As the technology matures and becomes more widely applied, it is expected to offer safer and more effective treatment options. However, further clinical research is crucial to validate these therapies’ effectiveness and address the current challenges and issues. Once these hurdles are overcome, stem cell treatments could revolutionize how we treat hair loss, providing long-term, sustainable solutions for patients.

#### Microneedling Technology

Microneedling technology has shown great potential in promoting hair regrowth by creating tiny punctures on the scalp (Fig. 9b), which helps improve the absorption of active ingredients and stimulates hair follicle regeneration [324]. This technique enhances the penetration of drugs and nutrients while activating the scalp’s natural healing mechanisms. When combined with growth factors and peptide ingredients, microneedling treatments have demonstrated significant hair growth-promoting effects in clinical settings [325]. Combining this technique with advanced formulations offers a promising approach to treating hair loss effectively and safely [326].

Microneedling is a minimally invasive skin treatment that uses fine needles to stimulate the skin’s surface, inducing collagen formation, generating new blood vessels, and producing growth factors [327]. Initially used for facial scars and skin rejuvenation, microneedling is now widely applied to various dermatological conditions [328], including androgenetic alopecia (AGA) and alopecia areata [329].

Studies show that microneedling can effectively combine with other hair growth therapies such as minoxidil, platelet-rich plasma (PRP), and topical steroids, enhancing hair follicle growth [330]. For example, one study demonstrated that microneedling-assisted human essential fibroblast growth factor (hbFGF) treatment significantly promoted hair growth in both male and female patients [331].

Additionally, microneedling can stimulate hair growth by enhancing transdermal absorption of drugs, increasing blood supply to hair follicles, stimulating dermal papilla cells and hair follicle stem cells, and activating the Wnt/β-catenin signaling pathway [332]. These combined mechanisms make microneedling a safe, practical, low-cost, and well-tolerated hair loss treatment option [333].

In clinical applications, microneedling has shown excellent therapeutic outcomes [334]. For example, a study involving microneedling combined with scalp nutrient solutions for AGA treatment significantly improved participants’ sebum secretion [335], scalp itching, dandruff, and hair loss. Another study explored the effects of combining microneedling with PRP therapy for AGA and found that this combination effectively improved follicular and hair density and enhanced scalp symptoms with excellent safety [336].

Therefore, microneedling technology enhances the absorption of drugs and nutrients and activates the scalp’s natural repair mechanisms. When combined with growth factors and peptides, microneedling therapy has demonstrated significant hair growth-promoting effects, offering a new treatment option for hair loss patients.

### Advanced Delivery Systems

Effective delivery systems ensure that anti-hair loss and hair growth products reach their full potential. Several advanced delivery systems have been developed to improve the penetration of drugs into the scalp, enhance absorption, and minimize side effects.

1. Microneedling Delivery System: This method involves creating tiny punctures in the scalp to enhance drug penetration. It directly delivers drugs to the hair follicles, significantly improving absorption efficiency. In addition, techniques such as laser-assisted, radiofrequency, and sonophoresis (ultrasound-based delivery) are used to enhance the scalp’s drug penetration capabilities further [337].
2. Nanotechnology in Drug Delivery: Nanoparticles are widely used as carriers in drug delivery systems [338]. By improving the solubility and stability of drugs, nanoparticles increase their absorption and distribution in the scalp [339]. For instance, novel lipid-based delivery systems like nanoemulsions and submicron emulsions can improve the water solubility of drugs, enhance their transdermal absorption, and thus increase their distribution and effect on the scalp [340].
3. Hair Follicle-Targeted Delivery Systems: Another advanced delivery technology focuses on directly targeting hair follicles. This system designs specific drug delivery carriers that act directly on the follicles, enhancing therapeutic outcomes while minimizing effects on other tissues [341]. For example, protein-lipid/polysaccharide composite delivery systems leverage the synergistic effects of proteins and polysaccharides to improve encapsulation efficiency and stability, maximizing the functionality and bioactivity of the product [342].

Therefore, microneedling, nanotechnology, and hair follicle-targeted drug delivery systems are advanced delivery systems currently used in anti-hair loss and hair growth products. These systems enhance the scalp penetration and absorption of drugs, improve treatment efficacy, and help minimize potential side effects. When selecting a delivery system, it is essential to consider the cause of hair loss and the targeted treatment area to ensure the best possible treatment outcomes.

#### Nanocarrier

Nanocarrier technology is an advanced drug delivery system that uses nanoparticles to encapsulate active ingredients, enhancing their stability and permeability. This technology is particularly effective for transdermal drug delivery systems, allowing drugs to pass through the skin barrier efficiently [343]. Using nanocarriers, the drugs can be directly delivered to the hair follicle area, increasing the local concentration of the drug and improving therapeutic outcomes.

Nanocarriers can significantly improve the solubility and bioavailability of active compounds, which is particularly beneficial for hydrophobic or poorly soluble drugs. This makes them ideal for treating conditions like hair loss, where localized delivery to the scalp is critical. Additionally, nanocarriers can reduce the risk of systemic side effects by targeting the drug directly to the treatment area.

Overall, nanocarrier technology offers a promising method for enhancing the effectiveness of anti-hair loss treatments, ensuring that active ingredients are delivered efficiently and with greater precision to the hair follicles, thus maximizing the therapeutic effects.

**Fig. 10:**
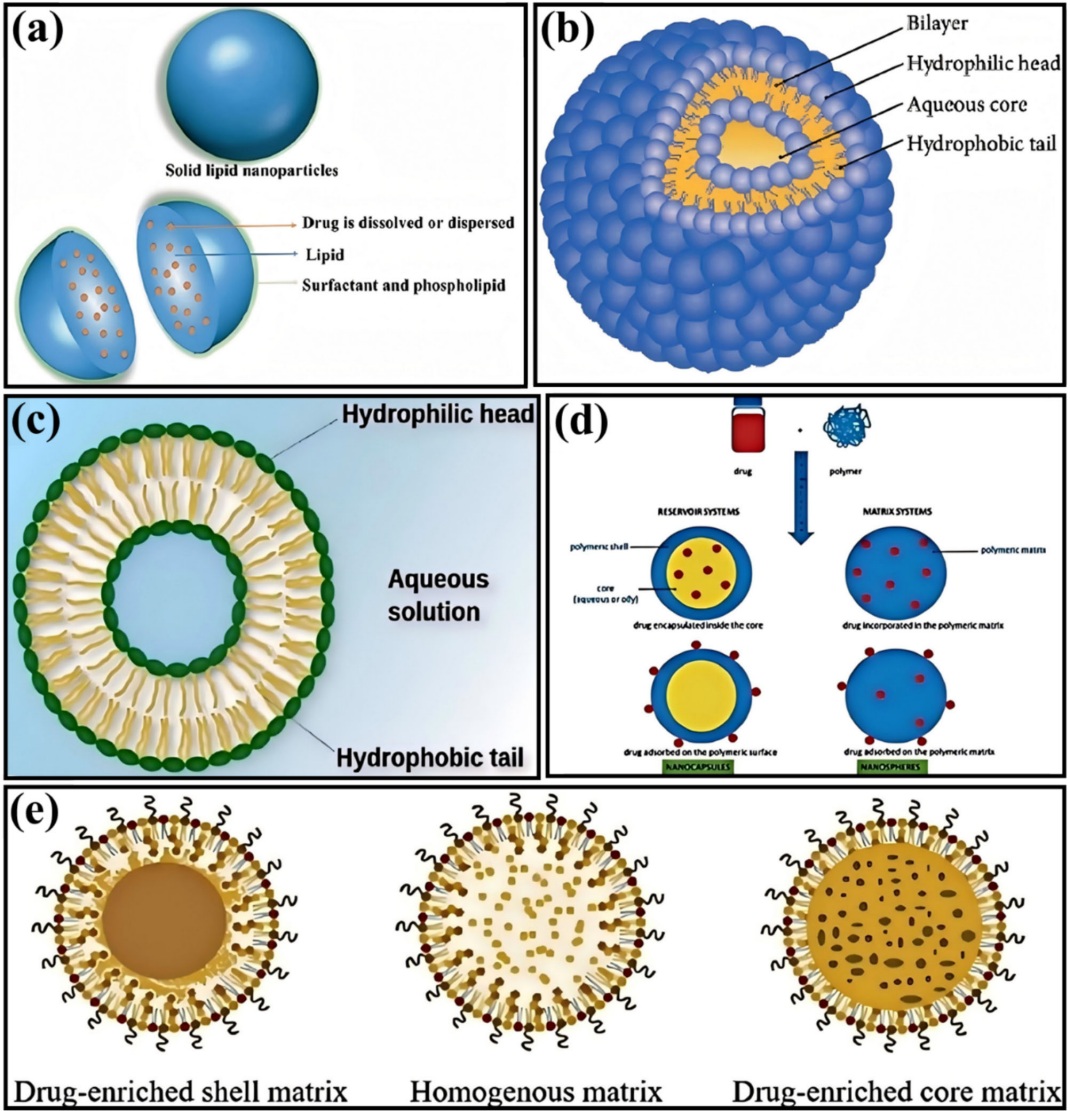
**a** Solid lipid nanoparticles as a drug delivery system. **b** Niosome structure [344]. **c** Scheme of a liposome formed by phospholipids in an aqueous solution [345]. **d** Solid lipid nanoparticles [346], **e** types of polymeric nanoparticles according to the composition [347]

Solid lipid nanoparticles (SLNs) (Fig. 10a), liposomes (Fig. 10b), and polymeric nanoparticles are three common forms of nanocarrier technology used for drug delivery systems [344]. Each nanocarrier has distinct properties and applications in enhancing drug delivery for hair growth treatments.

1. Liposomes are vesicles made of phospholipid bilayers that can encapsulate water-soluble (Fig. 10c) and lipophilic drugs. Liposomes are highly biocompatible and have low toxicity, making them ideal for delivering active ingredients to the scalp without causing adverse effects. They enhance the stability of the drugs and increase their absorption into the skin, which is crucial for effective hair follicle targeting [345].
2. Solid Lipid Nanoparticles (SLNs): SLNs (Fig. 10d) are composed of solid lipids, offering improved stability and controlled release of drugs. They help minimize side effects while maintaining a sustained release of active ingredients, ensuring the drug remains effective. This slow-release property can improve the overall treatment outcomes for hair regrowth by providing prolonged exposure to the active compound [346].
3. Polymeric Nanoparticles: These nanoparticles (Fig. 10e) are made from polymer materials that can encapsulate drugs in their core or adsorb them onto the shell. They improve drugs’ physical and chemical properties, protect them from premature degradation, and allow for precise control over the release kinetics. Polymeric nanoparticles offer flexibility in drug delivery, as they can be tailored to release their contents at specific rates, making them useful for targeted and effective hair loss treatments [347].

Each of these nanocarriers plays a crucial role in enhancing the bioavailability, stability, and controlled release of active ingredients, ensuring more effective hair loss and regeneration treatments.

**Fig. 11:**
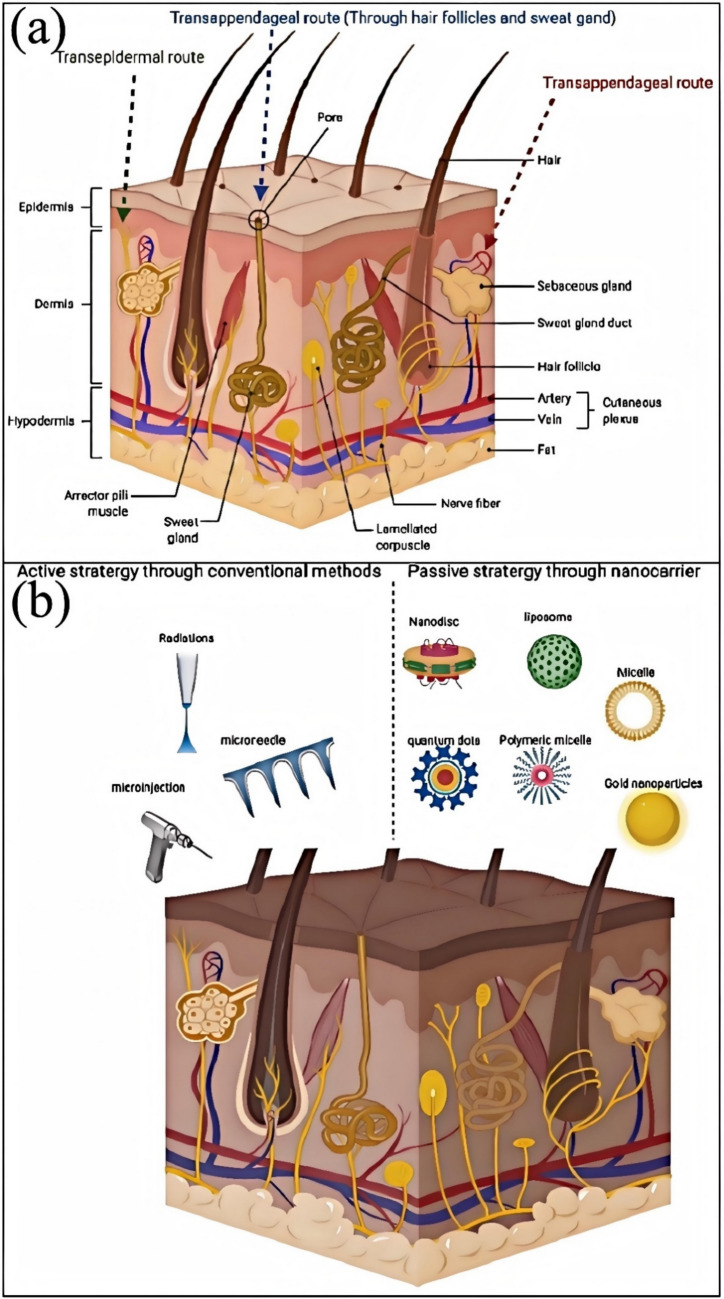
**a** Schematic illustration of the skin layer and showing penetration routes of the drug administered through the skin [348]. **b** Drug delivery strategy of nanocarriers and conventional approaches [349]

These nanocarriers enhance the skin permeability of drugs (Fig. 11a) and enable targeted drug delivery [348]. By encapsulating drugs within nanocarriers (Fig. 11b), it is possible to utilize specific targeting ligands or responsive mechanisms (such as pH or temperature sensitivity) to enhance drug accumulation in targeted areas of the skin. This targeted delivery ensures a higher concentration of the active ingredient at the desired site, increasing the treatment efficacy while minimizing systemic side effects [349].

For example, pH-responsive nanocarriers can release drugs specifically in areas with a specific pH, such as inflamed or diseased skin regions. Similarly, temperature-sensitive nanocarriers can release their contents in response to local heat generated by the skin, which can be especially useful in conditions where localized drug delivery is crucial. By combining these targeting strategies, nanocarrier-based systems significantly improve the precision and safety of treatments for hair loss, optimizing both the effectiveness and the patient’s experience.

**Fig. 12:**
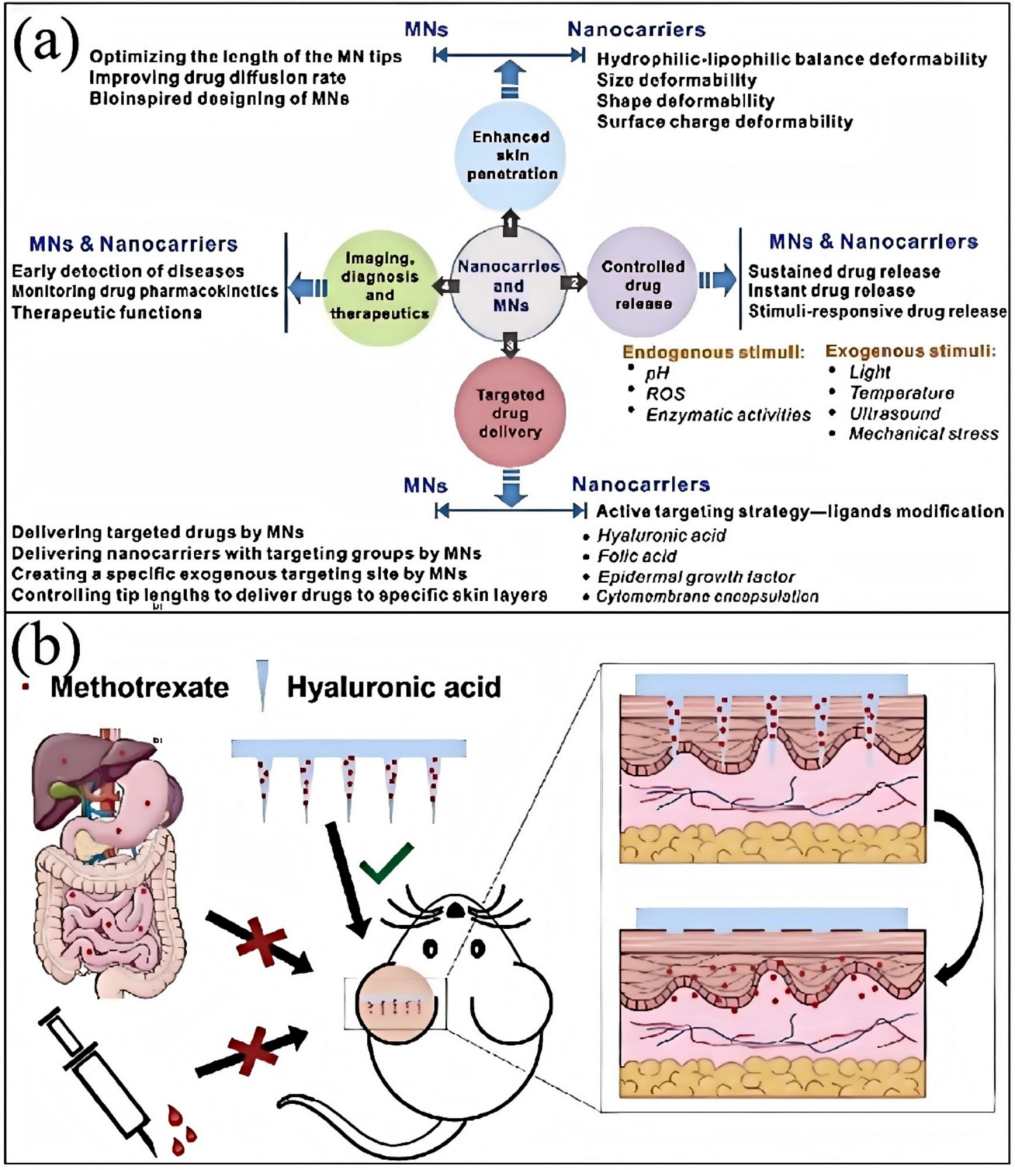
**a** Four categories of design principles based on nanocarrier and MN delivery systems with their representative examples [350]. **b** Schematic illustration of an HA-based DMN patch loaded with MTX to improve the treatment of psoriasis [351]

In addition, nanocarrier technology offers other advantages, such as the ability to deliver multiple drugs simultaneously (Fig. 12a), providing sustained release effects [350], and improving the solubility and stability of the drugs. These characteristics make nanocarriers an effective tool for treating various skin conditions, including but not limited to acne, psoriasis (Fig. 12b), eczema, and more [351].

By enabling the concurrent delivery of multiple active ingredients, nanocarriers can provide synergistic effects, enhancing the therapeutic outcomes for conditions that require a combination of treatments. For instance, in the case of psoriasis, nanocarriers can deliver anti-inflammatory agents alongside moisturizing compounds or other therapeutic substances, addressing multiple aspects of the condition in one treatment. The sustained release capability also ensures that the drug is gradually released over time, improving its effectiveness while minimizing side effects or the need for frequent application. These features make nanocarrier systems highly versatile and promising for dermatological applications, including hair loss treatments.

Therefore, nanocarrier technology, utilizing various forms of nanocarriers such as liposomes, solid lipid nanoparticles (SLNs), and polymeric nanoparticles, offers an efficient, safe, and targeted approach to transdermal drug delivery. The development of this technology not only enhances the therapeutic effects of drugs but also helps to reduce adverse reactions and improve patient compliance. However, despite the great potential of nanocarrier technology in treating skin conditions, further research and development are needed to realize its clinical applications fully. As this technology continues to evolve, it may revolutionize the treatment of various dermatological diseases, including hair loss, by improving drug efficacy and targeting specific areas for more precise and personalized therapy.

#### Transdermal Drug Delivery

**Fig. 13:**
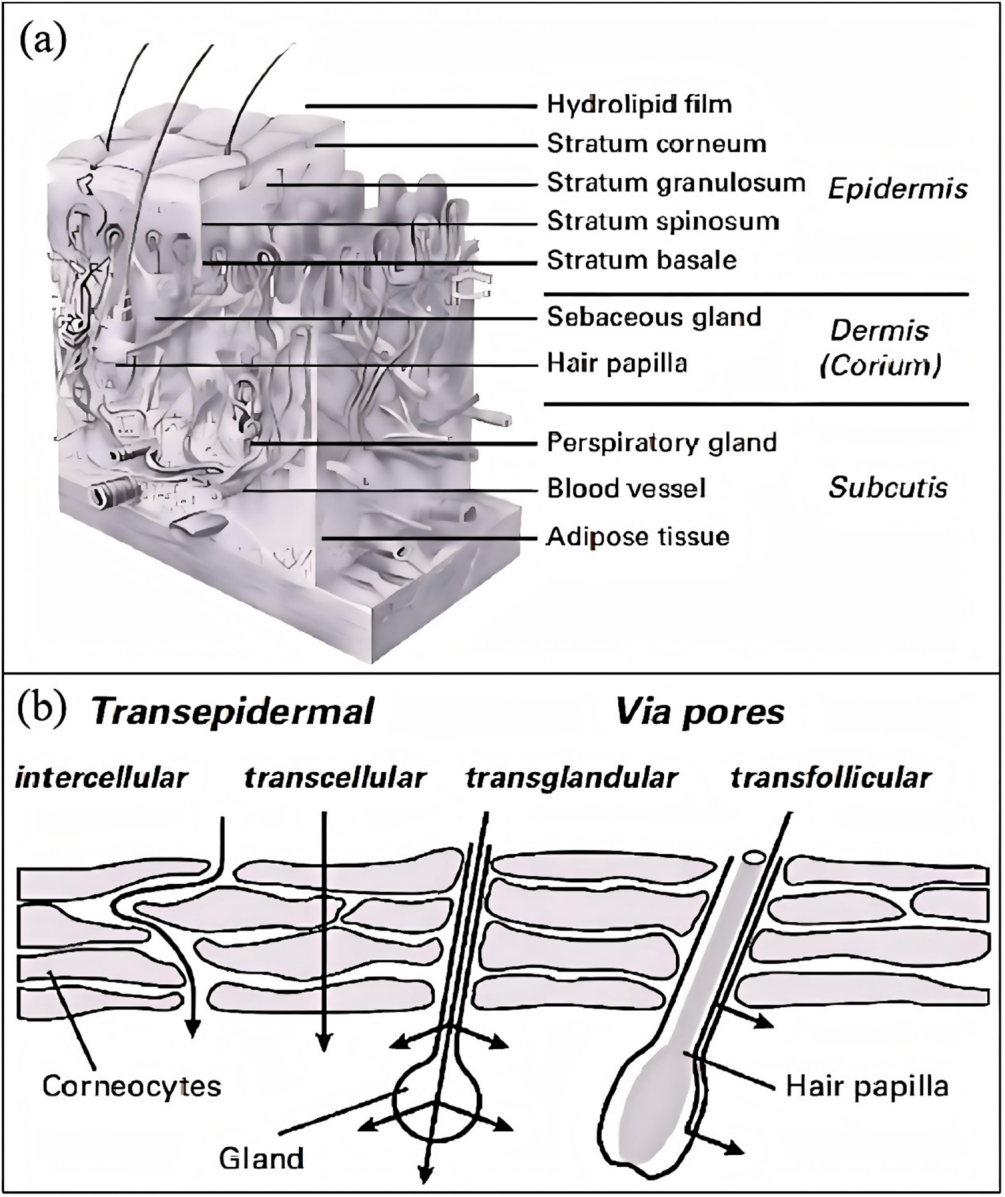
**a** The human skin schematically. Adapted from Skin Care Forum. **b** Options of drug penetration across the stratum corneum schematically [354]

Transdermal drug delivery systems (TTS) offer a non-invasive method of delivering medications directly through the skin into the bloodstream, effectively treating or preventing various conditions [352]. This delivery system provides advantages such as avoiding the first-pass metabolism in the liver, maintaining stable blood concentrations, local targeting, and convenience in administration [353]. However, as a biological barrier of the skin (Fig. 13a), the stratum corneum limits the permeation of many drugs, especially hydrophilic compounds (Fig. 13b) [354]. Researchers have developed various techniques to enhance skin permeability to overcome this barrier. These include transdermal enhancers, ultrasound, and electroporation, which help increase drug absorption and improve the system’s effectiveness. By employing such techniques, TTS has the potential to revolutionize the delivery of many therapeutic agents, including those for hair loss treatment, providing a more direct and efficient way of administering medication [355].

The application of nanotechnology has brought significant breakthroughs to transdermal drug delivery systems (TTS). Nanocarriers such as liposomes, polymer nanoparticles, and nanocrystals have been shown to enhance drug or vaccine permeability, control drug release, and target specific skin areas [356]. These nanocarriers not only improve the skin permeability of hydrophilic and hydrophobic drugs but also enable sustained and stable drug release, reducing the frequency of administration and increasing patient compliance.

**Fig. 14:**
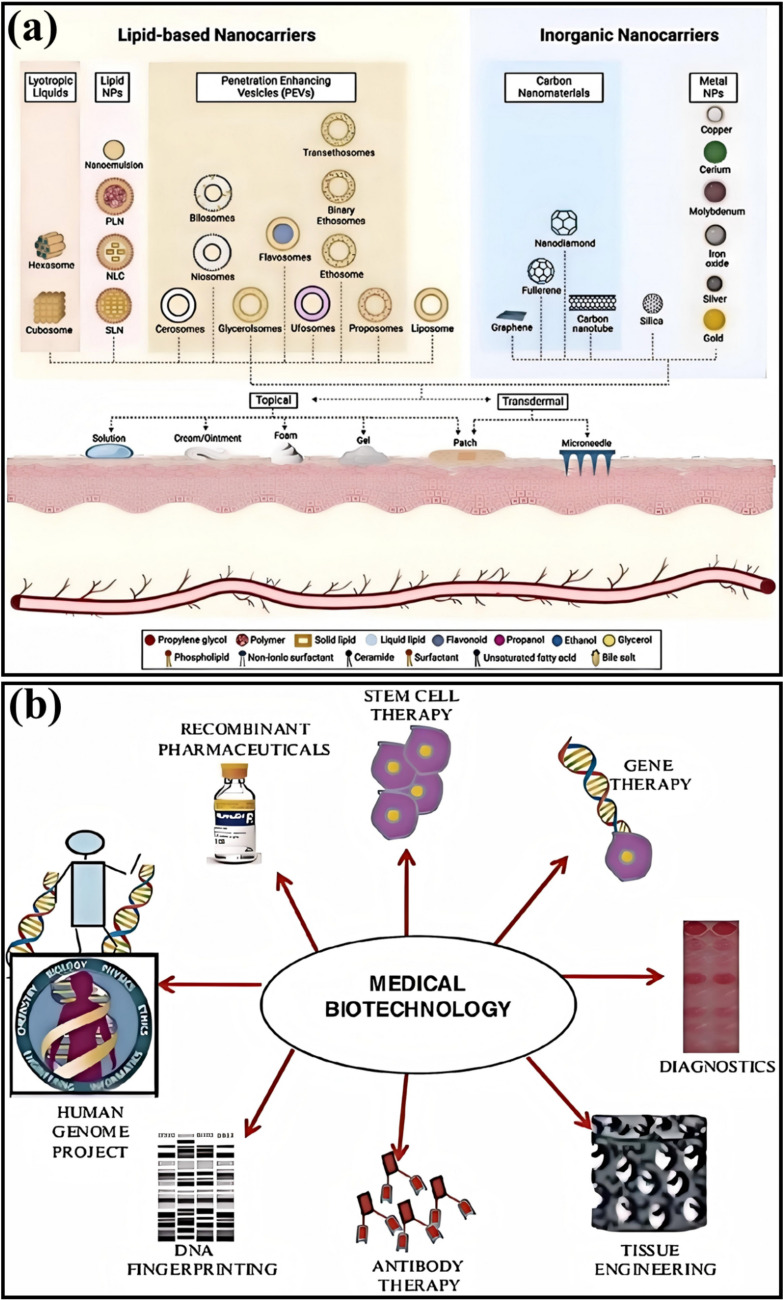
**a** Different types of nanocarriers for topical and transdermal drug delivery [353]. **b** Various applications of medical biotechnology [359]

In the treatment of hair loss and hair regeneration products, transdermal drug delivery systems also show tremendous potential. Due to the presence of the skin barrier, many drugs used for treating hair loss struggle to penetrate the skin effectively. By combining nanotechnology with transdermal drug delivery systems, the skin permeability of these drugs can be significantly improved, thereby enhancing their efficacy. For example, nanocarriers like nanoemulsions and lipid nanovesicles can effectively deliver drugs to hair follicles and other skin tissues (Fig. 14a), promoting hair growth [357]. This combination of nanotechnology and TTS offers a promising solution for more efficient and targeted treatments for hair loss.

Physical enhancement methods such as ultrasound and electroporation have also effectively increased drug permeability through the skin. Ultrasound permeation technology provides a sustained and stable permeation rate several times higher than conventional drug delivery methods. At the same time, electroporation creates transient, reversible, hydrophilic pores in cell membranes and skin using high-voltage pulses, thus enhancing molecular permeability [358]. Combining these physical enhancement methods with nanotechnology can further improve the efficiency and safety of transdermal drug delivery systems.

Therefore, transdermal drug delivery systems, through permeation enhancers, ultrasound, electroporation, and the integration of nanotechnology, can significantly improve the skin permeability of drugs, offering new strategies for treating hair loss and regeneration products. This integrated approach not only enhances drug efficacy but also reduces the frequency of administration and increases patient compliance, making it a promising and valuable new drug delivery method with great potential.

### Personalized Care

Personalised care is crucial in developing hair loss prevention and growth products. Combining genetic information with lifestyle factors can provide more precise and effective treatment plans for individuals.

Genetic factors are key to hair loss. Studies show that genetics and epigenetics closely interact with the root causes of hair loss [43] For example, TrichoTest™ provides individuals’ genetic profiles to help doctors avoid ineffective medications and focus on treatments more likely to produce positive results. Genetic information enhances medical treatment benefits while reducing the potential for related side effects.

Lifestyle factors also significantly influence hair loss. Diet, stress, and hormonal imbalances are lifestyle factors impacting hair health. For instance, poor lifestyle habits such as irregular eating patterns, late-night activities, and emotional fluctuations are common causes of hair loss in teenagers. Therefore, lifestyle changes like improving diet and reducing stress are crucial for preventing and treating hair loss.

Implementing personalized care requires considering both genetic and lifestyle factors [360]. For example, understanding an individual’s genetic predispositions and lifestyle habits allows for customized dietary and lifestyle recommendations to reduce the risk of hair loss or improve existing conditions. Personalized care may also include using specific scalp care products tailored to an individual’s needs.

Therefore, personalized care is a significant direction for the future of hair loss prevention and hair growth products. More targeted treatment plans can be developed by deeply understanding an individual’s genetic and lifestyle characteristics, ultimately improving treatment outcomes and patient satisfaction.

#### Genetic Testing and Personalized Formulations

Genetic testing technology can analyze an individual’s risk of hair loss and responsiveness to medications, providing personalized solutions for hair loss prevention and promotion of hair growth [361]. Based on genetic testing results, researchers can design formulations tailored to specific genotypes, maximizing treatment effectiveness. Additionally, personalized formulations can be adjusted according to an individual’s scalp condition and lifestyle habits, offering a comprehensive care plan.

This approach ensures that treatments are more effective and minimize the likelihood of side effects, as they are tailored to each person’s unique genetic makeup and lifestyle factors. With advancements in genetic testing, personalized hair care products are becoming a more promising direction for the future of hair loss treatment.

#### Application of Big Data and Artificial Intelligence

The application of big data and artificial intelligence (AI) in hair loss prevention and promotion of hair growth has immense potential [362]. By collecting and analyzing large amounts of data from hair loss patients, patterns and influencing factors can be identified, leading to the optimization of treatment plans. AI algorithms can recommend the most suitable products and care methods based on individual characteristics, improving the precision and effectiveness of treatment. In the future, the multidisciplinary collaboration will further advance the application of big data and AI in personalized care.

The use of artificial intelligence in the medical field, particularly in dermatology, is rapidly advancing. AI can now classify skin lesions accurately [363], and there have been emerging applications in hair restoration. For example, fully automated hair detection and growth measurement systems and scalp diagnostic systems based on deep learning and automatic hair loss measurement have been developed [364]. The development of these technologies opens up new possibilities for hair loss prevention and promoting hair growth.

Soon, integrating AI, big data, and personalized care will significantly enhance the accuracy and success of hair loss treatments.

Artificial intelligence (AI) is also used to develop hair follicle detection systems and biomedical products. These technologies analyze the scalp and hair texture to identify follicular issues and provide expert guidance more accurately. This application enhances diagnostic accuracy and creates permanent follicle health profiles for users by tracking services, recommending targeted treatments, and regularly pushing professional scalp care knowledge and product suggestions.

Machine learning techniques also show great potential in predicting hair health [365]. Studies have shown that random forest algorithms have achieved an accuracy rate of 94.6% in predicting hair health, highlighting the potential of machine learning in precise, non-invasive, and personalized hair health forecasting [366]. These findings suggest that hair care management can be further improved by integrating different data sources and developing user-friendly tools.

However, despite the broad prospects of AI in medicine, there are still challenges in practical clinical implementation. For example, the availability of high-quality, well-annotated dermatology datasets is a limiting factor, and significant technological investments and training for healthcare professionals are required. Moreover, issues around medical data privacy and the transparency of AI algorithms also need to be addressed.

Therefore, hair loss prevention and growth promotion products will achieve significant advances in efficacy, safety, and personalization through these innovative methods, meeting the growing market demand. In the future, as technology continues to develop and interdisciplinary collaboration strengthens, the application of big data and AI in personalized care will become more widespread and profound.

## Prospects

The hair loss prevention and growth cosmetics field is rapidly developing, and there is vast potential for future growth in technological advancements, market prospects, and interdisciplinary collaboration. This article provides an outlook to guide research, development, and application.

### Technological Development

1. Advances in Hair Growth Technologies: Recent research in hair growth technologies has mainly focused on applying natural plant extracts, mesodermal methods, and stem cell approaches [367]. These technologies show great potential due to their convenience, good targeting, fewer side effects, and cost-effectiveness. In addition, applying ingredients like hyaluronic acid in hair loss and growth cosmetics has garnered significant attention [368], offering new directions for cosmetic development through their moisturizing and skin-nourishing properties.
2. Efficacy Evaluation Methods: Efficacy evaluation methods for hair loss prevention products include in vitro, in vivo, and ex vivo testing [369]. These methods can be flexibly selected according to the situation to assess the effectiveness of hair loss products visually. Furthermore, high-precision analytical techniques, such as ultra-high-performance liquid chromatography-quadrupole-time-of-flight mass spectrometry, provide powerful tools for screening and quantitatively analyzing illegal chemical additives in hair growth cosmetics.

### Market Prospects

1. Growing Market Demand: As living standards improve and individuals place more importance on their image, the demand for hair loss prevention and hair growth products continues to grow. The demand for products with hair loss prevention and growth-promoting effects is strong and expected to generate substantial profits for manufacturers. Moreover, different hair growth cosmetics, including herbal-based, chemical-based, and biochemistry-based products, are becoming popular, indicating a diversified market demand.
2. Differentiated Competition: To meet market demand, products such as anti-hair loss shampoos must adopt a differentiated approach [370]. By profoundly analyzing market trends, manufacturers must continuously innovate and develop unique efficacy and safety products to cater to personalized consumer needs.

### Interdisciplinary Collaboration

1. Cross-Disciplinary Research: Developing hair loss prevention and growth cosmetics requires collaboration across multiple disciplines, including biology, chemistry, pharmacology, and materials science. For example, by studying the biological characteristics of hair follicles and the pathogenesis of hair loss, experts can better understand the mechanisms of active ingredients, leading to the development of more effective products for hair loss prevention and growth [371].
2. Regulation and Safety Oversight: As the market for hair loss cosmetics rapidly expands, the importance of regulation and safety oversight becomes increasingly significant. Establishing an efficient safety monitoring system to ensure that products do not contain harmful ingredients can boost consumer trust in hair loss prevention cosmetics and foster healthy market growth.

The hair loss prevention and growth cosmetics sector is in a period of rapid development, with considerable potential for future progress in technological innovation, market demand, and interdisciplinary cooperation. Through ongoing technological advancements and market research, combined with collaborative efforts across various fields, more effective and safer products can be developed to meet consumer needs and promote the industry’s sustainable growth.

### Technology Development Trends

The future development trends in hair loss prevention and growth technologies will focus on achieving efficient, safe, personalized treatment options. Advancements in gene therapy, stem cell technology, microneedling, advanced delivery systems, and the application of artificial intelligence and big data analysis will be the key factors driving progress in this field.

Gene therapy and stem cell technologies have already shown significant potential in hair loss treatment. For example, adipose-derived stem cells are emerging as a novel therapy for hair loss due to their ability to regulate the hair follicle cycle and promote angiogenesis [372]. Furthermore, research on stem cell therapy for androgenic alopecia (AGA) has shown that stem cells play a role in tissue repair and maintaining the homeostasis of the microenvironment, offering new strategies for treating AGA [373]. These studies suggest that stem cell technology is broadly applied in hair loss treatment, particularly in regenerative medicine and hair tissue engineering.

In addition to gene therapy and stem cells, advancements in microneedling and drug delivery systems are expected to enhance the effectiveness of hair loss treatments by improving drug absorption and targeting. Advanced systems, including nanoparticle-based delivery and hair follicle-targeted approaches, will likely provide more precise and controlled therapies. Artificial intelligence and big data analytics will further enhance personalized care, enabling clinicians to tailor treatments based on individual genetic profiles and lifestyle factors, optimizing outcomes for each patient.

Therefore, developing high-efficiency, safe, and personalized treatments in hair loss prevention and growth will be propelled by ongoing advances in gene therapy, stem cell technology, delivery systems, and the integration of artificial intelligence and data analysis. These innovations promise to revolutionize hair loss treatments, making them more effective and accessible for a broader range of individuals.

The development of microneedling technology and advanced delivery systems has significantly enhanced the absorption and effectiveness of active ingredients by promoting transdermal absorption and improving blood supply to hair follicles. Studies on microneedling-based treatments for female pattern hair loss have shown that this technique effectively promotes hair regrowth, inhibits hair loss, improves dermoscopic signs, and even enhances patients’ quality of life and alleviates negative emotions.

The application of artificial intelligence and big data analysis will further optimize personalized treatment plans, increasing the accuracy and effectiveness of treatments. By analyzing large volumes of clinical data and patient information, AI can more precisely predict treatment outcomes, enabling healthcare providers to offer more tailored solutions for individuals.

The continuous advancements in gene therapy, stem cell technology, microneedling, advanced delivery systems, and the integration of AI and big data analysis will collectively drive hair loss prevention and hair growth technologies toward more efficient, safe, and personalized treatments. These innovations promise breakthroughs in hair loss treatment and enhance the precision and effectiveness of these therapies, offering greater hope and options for patients suffering from hair loss.

### Market Outlook Prediction

With the increasing prevalence of hair loss and the growing importance of people’s appearance, the market for hair loss prevention and hair growth promotion products will continue to grow [374]. This trend is reflected globally, especially in emerging markets such as China and India, where market demand will rapidly expand with improved living standards and enhanced purchasing power. Efficient, safe, and personalized products will become the mainstream in the market, catering to the diverse needs of consumers.

Progress has been made in the research and market promotion of hair loss prevention and hair growth products. For instance, research on compound plant ingredients has shown that combining ginger juice, polygonum multiflorum, and angelica extract can effectively inhibit 5α-reductase activity and promote hair growth. In addition, biota leaves and polygonum multiflorum extracts also show significant anti-hair loss effects. These research findings provide a scientific basis for developing hair loss prevention products.

Market analysis shows that the market size for anti-hair loss shampoos is continually growing. In 2018, the market size for anti-hair loss shampoos in China reached 1.13 billion yuan, expected to exceed 1.5 billion yuan by 2020. This data indicates strong market demand and commercial potential for anti-hair loss products [375].

However, the market also faces some challenges. For example, M company’s H brand anti-hair loss shampoo has mediocre performance in the Chinese market. The reasons include insufficient brand awareness, significant regional advantages of competitors, and the existing marketing strategy’s inability to gain a competitive edge. This indicates that while there is market demand, companies must adopt more effective marketing strategies and product innovations to meet the market’s needs.

Moreover, consumer preferences for anti-hair loss products are also changing. A survey in the Guangxi Zhuang Autonomous Region revealed that the demand for anti-hair loss and hair growth products among young people is increasing. Still, relevant companies lack a proper understanding of consumer needs. This suggests that companies must pay more attention to consumer demands, segment the market, and develop personalized products.

The global market for hair loss prevention and hair growth promotion is expected to continue growing, especially in emerging markets like China and India. Companies should focus on the changing needs of consumers, develop efficient, safe, and personalized anti-hair loss products, and adopt effective marketing strategies to enhance brand awareness and market competitiveness. At the same time, continuous investment in research and development and innovation is key to maintaining market competitiveness.

### The Necessity of Multidisciplinary Collaboration

The research and development of hair loss prevention and hair growth promotion is a complex process that requires multidisciplinary collaboration to achieve technological breakthroughs and promote the application of innovations. Integrating fields such as medicine, beauty, biotechnology, and materials science has provided new ideas and methods for product innovation and optimization.

From a medical perspective, hair loss is closely related to genetics, aging, immunity, hormones, infections, and mental and psychological aspects. Therefore, medical research plays a significant role in preventing hair loss and promoting hair growth. By studying the anti-hair loss and hair growth effects of traditional Chinese medicine and its active ingredients, as well as their mechanisms, new theories and approaches for preventing and treating hair loss have been developed. In addition, the application of neurobiology has also provided a multifaceted approach to solving hair-related problems [376].

In the beauty sector, the development of the cosmetics industry and technology has driven the innovation of hair loss prevention and growth products. For example, ginger extract has been widely used in products due to its strong historical applications and claimed efficacy [377]. Furthermore, photodynamic energy hair growth and care technology has demonstrated excellent scientific research value, offering protection for hair after transplantation [378].

Biotechnology is also widely applied in hair loss prevention and hair growth promotion. For instance, multi-omics joint analysis has been used to identify probiotics that can repair hair damage, laying a theoretical foundation for developing safer and more effective foods and drugs. Additionally, high-pressure homogenization technology has been employed to prepare composite nanoliposomes containing active ingredients from different mechanisms of action, showing promising application prospects.

Materials science also plays an essential role in developing hair loss prevention and hair growth products. For example, by optimizing extraction processes, efficient preparation of volatile oils from ginger plants has been achieved, providing direction for related product development [379]. Furthermore, research on the human hair growth and care photodynamic energy system has solved the technical challenge of uneven light emission by designing and optimizing key parameters in the lighting system.

Researching and developing hair loss prevention and hair growth promotion requires the collaboration of disciplines such as medicine, beauty, biotechnology, and materials science. By combining these fields, new ideas and methods for product innovation and optimization can be provided, leading to technological breakthroughs and the widespread application of these advancements.

#### The Combination of Medicine and Beauty

Combining medicine and beauty in treating hair loss can address the problem from internal and external perspectives. Medical research provides an in-depth understanding of the mechanisms of hair loss, helping to develop more effective treatment plans. At the same time, beauty technologies can improve the scalp environment, promote drug absorption, and support healthy hair growth.

From a medical perspective, treatments for hair loss include various methods such as drug therapy, surgical treatments, and a combination of traditional Chinese medicine and Western medicine. Minoxidil and finasteride are commonly used drug treatments that slow down or prevent the progression of hair loss through different mechanisms. In addition, the combination of traditional Chinese and Western medicine for treating alopecia has shown promising results. This approach combines the holistic treatment of traditional Chinese medicine with the local treatments of Western medicine, offering a more comprehensive solution to hair loss.

From the perspective of beauty technologies, scalp tattooing and microneedling techniques promote hair growth by improving the scalp environment. Scalp tattooing works by adding pigments to the scalp to conceal areas of hair loss, thereby improving appearance. Microneedling stimulates the scalp, promotes blood circulation, and enhances drug absorption, accelerating hair growth [380]. These techniques improve the patient’s appearance and directly affect the scalp through physical or chemical means, promoting hair growth.

Combining medicine and beauty provides multi-angle, multi-level solutions for treating hair loss. Medical treatments address the root causes of hair loss, while beauty technologies focus on improving the scalp environment and appearance. Combining the two can significantly enhance the overall effectiveness of hair loss prevention and hair growth promotion.

#### The Integration of Biotechnology and Material Science

Integrating biotechnology and materials science is key to developing hair loss prevention and growth promotion products. Biotechnology provides the foundation for developing new active ingredients and treatment methods, such as gene editing, stem cell technology, and fermentation engineering [381]. These technologies enable the synthesis of safer and more cost-effective materials than traditional chemical materials and allow for industrial-scale production through techniques like synthetic biology. For example, recombinant humanized collagen has been used as an ingredient in skincare products, demonstrating the potential application of biotechnology in the cosmetics industry [382].

Materials science supports the innovation of delivery systems, such as nanocarriers and transdermal drug delivery technology [383]. The application of nanotechnology has expanded the range of drugs that can be delivered transdermally and improved the therapeutic effects of these drugs. For instance, as a novel drug delivery method, nanoliposomes can significantly enhance the transdermal absorption rate of active ingredients and ensure cellular safety. Moreover, research advancements in the application of nanodevices in transdermal drug delivery systems show that nanotechnology can overcome specific barriers in biopharmaceutical production, such as the toxicity risks of water-soluble/insoluble drugs and cosmetic ingredients. It also increases bioactivity, specificity, tolerance, and therapeutic indices.

Integrating biotechnology and materials science through interdisciplinary collaboration can deliver active ingredients precisely, improve product stability and safety, and ultimately enhance therapeutic outcomes. For example, by using high-pressure homogenization technology to prepare active ingredients with different mechanisms into composite nanoliposomes (Fig. 14b), not only is the transdermal absorption rate of active ingredients increased, but it also significantly reduces the oxidative damage of hydrogen peroxide to dermal papilla cells, reverses the decrease in mitochondrial membrane potential, and inhibits cell apoptosis [359]. Additionally, research on composite extracts of biota leaves and polygonum multiflorum shows that these extracts have anti-hair loss effects and can be used to develop personal care products for hair loss prevention.

In the future, the anti-hair loss and hair growth cosmetics field will make significant progress in technological innovation and market expansion [384]. Multidisciplinary cooperation will provide new momentum for research and development, driving products toward greater efficiency, safety, and personalization. Through continuous innovation and optimization, anti-hair loss and hair growth products will better meet market demands and enhance consumers’ quality of life.

## Conclusion

The field of anti-hair loss and hair growth-promoting cosmetics has demonstrated considerable research and application potential in addressing the increasingly severe problem of hair loss. The physiological and pathological mechanisms of hair loss are complex and diverse, involving types such as androgenetic alopecia, resting phase alopecia, and alopecia areata, and are influenced by multiple factors such as genetics, environment, and lifestyle. A wide variety of existing products, including minoxidil, finasteride, plant extracts, growth factors, and peptides, have been developed to address this issue. Nevertheless, challenges persist with regard to the effectiveness, safety, consumer acceptance, and complexity of regulatory standards. The continuous emergence of innovative technologies, including gene therapy, stem cell technology, microneedle technology, advanced delivery systems, and personalized care solutions, has resulted in a paradigm shift in the field, with interdisciplinary collaboration now being identified as a key driving force for its rapid development. Future research should further reveal the molecular and genetic mechanisms of hair loss, optimize existing treatment plans, develop personalized and precise treatment strategies, and explore the potential of new active ingredients. At the industry level, investment in scientific research should be increased, regulatory standards improved, consumer education enhanced, and market standardization and international cooperation promoted. Through multi-party collaboration, the cosmetics industry for preventing hair loss and promoting hair growth is expected to usher in broader development prospects, providing safer, more effective, and personalized solutions for hair loss patients.

## Data Availability

Not applicable.
